# LILRB1 Is a Prognostic‐Related Biomarker Correlated With Immune Infiltration in Head–Neck Squamous Cell Carcinoma

**DOI:** 10.1002/cam4.71727

**Published:** 2026-03-25

**Authors:** Shuai Chen, Qiuwan Wu, Shuo Gu, Yi Zhou, Mingquan Cai, Junhua Wu, Jing He, Jingjing He, Juli Lin, Zhicong Hong, Binghuang Zhang, Xianyang Luo

**Affiliations:** ^1^ Department of Otolaryngology‐Head and Neck Surgery, the First Affiliated Hospital of Xiamen University, School of Medicine Xiamen University Xiamen People's Republic of China; ^2^ Xiamen Key Laboratory of Otolaryngology Head and Neck Surgery Xiamen China; ^3^ Department of Scientific Research, the First Affiliated Hospital of Xiamen University, School of Medicine Xiamen University Xiamen People's Republic of China; ^4^ The Third Clinical Medical College Fujian Medical University Xiamen Fujian People's Republic of China; ^5^ Shenyang City University Shenyang City China; ^6^ Medical Oncology, the First Affiliated Hospital of Xiamen University, School of Medicine Xiamen University Xiamen People's Republic of China; ^7^ Department of Breast Surgery, Women and Children's Hospital, School of Medicine Xiamen University Xiamen Fujian Province China

**Keywords:** CXCL13, head and neck squamous cell carcinoma, immune effector cell, immune infiltration, LILRB1, SPP1‐ACP5^+^ macrophage

## Abstract

**Background:**

Conventional treatment strategies and immunotherapy yield low response rates in head and neck squamous cell carcinoma (HNSCC). This study aimed to explore the potential of immunosuppressive receptor leukocyte immunoglobulin‐like receptor subfamily B member 1 (LILRB1) for developing effective immunotherapies for HNSCC.

**Methods:**

Clinical data collection and analysis were determined using Tumor Immunity Estimation Resource Database (TIMER) and Cancer Treatment Response gene signature DataBase (CTR‐DB). Immunohistochemistry was used to detect protein level in fresh breast cancer tissues. RNA sequencing was employed to screen the downstream signaling pathway in WBP2‐overexpressed cells. Tumor xenograft model and Flow Cytometry were performed to monitor tumor growth and cell apoptosis, respectively.

**Results:**

In this study, high expression of LILRB1 in HNSCC tissues was observed compared to normal tissues. HNSCC patients with high LILRB1 expression exhibited a better prognosis, which was influenced by tumor mutation burden. Functional network analysis revealed a positive association between LILRB1 and chemokine signaling pathways. Copy number variation of LILRB1 was positively correlated with the infiltration of CD8+ T cells and M1 macrophages. The prognostic effect of LILRB1 depends on CD8+ T‐cell abundance, especially in HNSCC with a low neoantigen load. High LILRB1 expression was associated with a higher immune score, indicating favorable outcomes in HNSCC patients receiving immunotherapy. Notably, LILRB1 was specifically expressed in SPP1‐ACP5+ macrophages; in these cells, high LILRB1 might reduce the proportion of cancer cells via the SPP1‐CD44 axis, and subsequently regulate CD8+ T cell enrichment through the C‐X‐C motif chemokine 13 (CXCL13)‐CXCR5 axis.

**Conclusion:**

Collectively, high LILRB1 expression in HNSCC was accompanied by the infiltration of various immune effector cells. LILRB1 may shape the tumor microenvironment of HNSCC, mediating the interactions between cancer cells and macrophages, as well as between cancer cells and CD8+ T cells, via the SPP1‐CD44 and CXCL13‐CXCR5 axes. Our findings illustrate that LILRB1 serves as a prognostic‐related biomarker associated with immune infiltration in HNSCC.

**Abbreviations**
CAMOIP: Comprehensive Analysis on Multi‐omics of Immunotherapy in Pan‐cancer
CXCL13: C‐X‐C motif chemokine 13
DCs: dendritic cells
ENCORI: Encyclopedia of RNA Interactomes
GEPIA2: Gene Expression Profiling Interactive Analysis 2
HNSCC: head and neck squamous cell carcinoma
ICBatlas: Immune Checkpoint Blockade Therapy Atlas
KM: Kaplan–Meier
LILRB1: leukocyte immunoglobulin‐like receptor subfamily B member 1
SCTIME: single cell transcriptomes of tumor immune microenvironment
TIMER: Tumor Immunity Estimation Resource Database
TISIDB: tumor‐immune system interactions database
TMB: tumor mutational burden
TME: tumor microenvironment

## 1 Introduction

Head and neck squamous cell carcinoma (HNSCC) accounts for over 90% of all head and neck cancers [1]. HNSCC arises from the mucosal epithelium of the oral cavity, pharynx, and larynx and ranks as the sixth most prevalent cancer globally [2]. Precancerous lesions are rarely detected, and most HNSCC patients are diagnosed at an advanced stage initially with a high propensity for local progression and relapse within 3 years [3]. Conventional surgery combined with radiotherapy and chemotherapy remains the primary therapeutic approach for HNSCC patients [4]. However, conventional therapeutic strategies have failed to significantly improve the cure rate of HNSCC over the past decade. Thus, the diagnosis and treatment of HNSCC remain a major challenge in clinical practice.

HNSCC is characterized by an immunosuppressive tumor microenvironment (TME) that evades immune surveillance via multiple resistance mechanisms. The TME of HNSCC consists of many different cell subsets, including cancer‐associated stromal fibroblasts, T cells, B cells, neutrophils, macrophages, myeloid‐derived suppressor cells (MDSCs), natural killer (NK) cells, and mast cells [5]. The infiltration of distinct cell subsets and their crosstalk through diverse signaling networks play a crucial role in mediating immune surveillance and regulating tumor growth [6]. In fact, tumor progression is driven by certain immune cell subsets (e.g., MDSCs and M2 macrophages) that promote tumor growth; in addition to that, tumors frequently evade host immune surveillance by suppressing cytotoxic T cell function or by activating and expanding immunosuppressive cell populations [7]. Immunotherapy has emerged as a promising strategy in recent years, with the development of diverse immunotherapeutic approaches. The burgeoning immune checkpoint blockades (ICB), such as anti‐CTLA‐4, anti‐programmed death 1 (PD‐1), and anti‐programmed death‐ligand 1 (PD‐L1) antibodies, in combination with immune stimulation, have yielded favorable outcomes in patients with recurrent or metastatic HNSCC [8]. Unfortunately, the response rate to ICB remains below 20% in patients with recurrent or metastatic HNSCC [9, 10]. Thus, there is an urgent need for novel strategies to develop more effective immunotherapies for HNSCC patients, with the aim of inhibiting HNSCC tumorigenesis and progression.

LILRB1 (Leukocyte Immunoglobulin‐like Receptor subfamily B member 1) is a member of the Leukocyte Immunoglobulin‐like Receptor B family. The LILRB family comprises LILRB1, LILRB2, LILRB3, LILRB4, and LILRB5; most of them are expressed on the surface of immune cells and bind to their cognate ligands to recruit and transduce intracellular inhibitory signals, leading to impaired immune cell activity [11]. LILRB1, also known as immunoglobulin‐like Transcript 2 (ILT2), CD85J, or LIR1, is a type I transmembrane glycoprotein with four extracellular immunoglobulin‐like domains. It can bind to specific ligands, including major histocompatibility complex (MHC) Class I (MHC‐1) [12], UL18 [13], S100A8, and S100A9 [14]. Upon interaction of LILRB1 with its ligands, intracellular immunoreceptor tyrosine inhibitory motifs are activated to modulate immune cell activity [15]. LILRB1 is widely expressed on NK cells, mononuclear macrophages, dendritic cells (DCs), T cells, and B cells [16, 17]. Studies have demonstrated that the MHC‐1/LILRB1 interaction can promote the expansion of MDSCs, thereby impairing the proliferative and cytotoxic functions of T cells [18, 19]. The MHC‐1/LILRB1 interaction also inhibits NK cell polarity and cytotoxicity [20]. However, the role of LILRB1 in the TME and HNSCC progression remains largely unexplored.

In this study, highly expressed LILRB1 was observed in HNSCC tissues, and this expression was positively associated with favorable clinical outcomes in HNSCC patients. LILRB1 primarily modulates HNSCC progression by regulating cytokine‐cytokine receptor interactions. Single‐cell RNA sequencing analysis was utilized to delineate the immune landscape and tumor heterogeneity in a cohort of HNSCC patients with varying LILRB1 expression. These findings reveal a potential crosstalk between LILRB1 and the TME, offering valuable insights for the development of immunotherapies for HNSCC.

## 2 Materials and Methods

### 2.1 Data Collection

Multiple independent public datasets including Encyclopedia of RNA Interactomes (ENCORI) database, Tumor Immune Estimation Resource (TIMER), Kaplan–Meier (KM), Tumor‐immune system interactions database (TISIDB), LinkedOmics, Comprehensive Analysis on Multi‐Omics of Immunotherapy in Pan‐cancer (CAMOIP), Single cell transcriptomes of tumor immune microenvironment (SCTIME), Immune Checkpoint Blockade therapy Atlas (ICBatlas) and Gene Expression Profiling Interactive Analysis 2 (GEPIA2) were utilized in this study.

### 2.2 Expression and Association Analysis

The ENCORI and TIMER databases were utilized to analyze transcriptional expression of LILRB1 in HNSCC and normal samples. The differential expression of LILRB1 in HNSCC patients who received immunotherapy was analyzed using the TISIDB database and receiver operator characteristic (ROC) curves, respectively. Differentially expressed genes (DEGs) in HNSCC patients were analyzed and identified using the CAMOIP and ICBatlas databases. Association analyses were performed using the GEPIA2 database. LILRB1 expression across different cell populations was retrieved from the SCTIME database. The KM Plotter (follow up threshold: 240 months) and ROC curves were used to analyze overall survival (OS) and relapse‐free survival (RFS) of HNSCC patients.

### 2.3 RNA Extraction and Quantitative Real‐Time PCR Analysis (qRT‐PCR)

Fresh HNSCC samples (*n* = 35) were collected and lysed using TRIzol RNA extraction reagent. Paracancerous tissues were collected from the mucosal tissue 2 cm away from the edge of the primary hypopharyngeal carcinoma lesion. These tissues were confirmed to be free of squamous cell carcinoma cells and infiltrating inflammatory cells by clinical pathologists via hematoxylin–eosin (HE) staining. cDNA was synthesized using ReverTra Ace (Toyobo, Osaka, Japan). qRT‐PCR was performed using SuperScript II (Invitrogen) according to the manufacturer's instructions. The primer sequences for LILRB1 were as follows: forward, 5′‐GGACACTCGGAGCCCACACG‐3′; reverse, 5′‐CAGCTCCCATGCATTCCAGACTCC‐3′. The amplification protocol was as follows: 50°C for 2 min, 95°C for 10 min, 40 cycles of 95°C for 15 s and 60°C for 1 min. GAPDH expression was used as an endogenous reference gene. Relative gene expression was calculated using the 2^−ΔΔ*CT*^ method.

### 2.4 Immumohistochemical Staining (IHC)

Fresh HNSCC tissue samples (*n* = 35) were obtained from The First Affiliated Hospital of Xiamen University (Xiamen, China). These samples were derived from independent patient cohorts. All experiments were approved by the Medical Ethics Committee of the First Affiliated Hospital of Xiamen University (XMYY‐2022KYSB008). Written informed consent was obtained from all patients. The study was conducted from January 2022 to March 2025. Tissue sections were dewaxed and rehydrated, followed by antigen retrieval using a high‐pressure steaming method. Endogenous peroxidase activity was blocked with 3% H_2_O_2_. After blocking with 10% bovine serum albumin (BSA) for 1 h at room temperature, the sections were incubated with a primary antibody against LILRB1 (26455‐1‐AP, Proteintech, US) at 4°C overnight. The next day, the sections were incubated with HRP‐conjugated secondary antibodies at room temperature for 1 h. The slices were visualized with DAB (ZL1‐9081, ZSGB‐BIO, China) following DAPI counterstaining, and images were captured using an inverted biological microscope (IX51, Olympus, Japan).

### 2.5 LinkedOmics Analysis

The LinkedOmics database (http://www.linkedomics.org) was utilized to analyze clinical data of HNSCC patients. The LinkFinder module was used to identify DEGs correlated with LILRB1 in the HNSCC cohort, and the results were visualized via heatmaps. Distinct signaling pathways and molecular networks were identified using the LinkInterpreter module. Gene set enrichment analysis (GSEA) was performed to annotate GO (Cellular Component: CC; Biological Process: BP; and Molecular Function: MF) and KEGG pathways. The parameters were set as follows: false discovery rate (FDR) < 0.05, with 500 permutations performed based on the rank criterion.

### 2.6 Immune Cell Infiltration Analysis

The association between the copy number variation (CNV) and the abundance of tumor‐infiltrating immune cells (TIICs) including B cells, CD4^+^ T cells, CD8^+^ T cells, neutrophils, macrophages, and dendritic cells, as well as the correlation between LILRB1 expression and TIICs abundance were analyzed using the TIMER database. TIICs infiltration profiles were analyzed using the CAMOIP database. Immune cell scores and the association between tumor mutational burden (TMB) and LILRB1 expression were monitored using the CAMOIP database.

### 2.7 Single‐Cell Analysis

The SCTIME database was utilized to analyze the differential expression of LILRB1 across immune cell subsets, construct an interaction network between macrophages and cancer cells, and assess the association between LILRB1 expression in SPP1‐ACP5^+^ macrophages and AREG^+^ macrophages and cancer cell abundance. Briefly, the expression matrix of the GSE103322 dataset was downloaded from the GEO database. Cells isolated from 18 patients were profiled via the Smart‐seq2 protocol and aligned to the human reference genome (hg19, version 19). Ultimately, the dataset included expression data for 5902 individual cells on 23,669 genes. The “FindVariableFeatures” function was employed to select the top 2000 most highly variable genes. The dataset was analyzed using the Seurat (3.1.5) package in R (v4.3.1). Cell clustering was performed using the “RunUMAP” function (dims = 1:10) for non‐linear dimensionality reduction. The “CalculateProportion” and “CalculateCorrelation” functions were use to compute the proportion of each cell type per patients and the correlation between distinct cell types, respectively. Cell type annotation was based on a previous study [21]. Among all cells, SPP1‐ACP5^+^ macrophages accounted for an average of 3.2% (Figure S1).

### 2.8 Cell‐to‐Cell Communications Analysis

Cell‐to‐cell interaction was analyzed using the CellChat (v1.1.3) algorithm to infer interactions between SPP1‐ACP5^+^ macrophages and cancer cells, based on data from the SCTIME database. The expression distributions of LILRB1, SPP1, ACP5, C‐X‐C motif chemokine 13 (CXCL13), CXCR5, and CD44 across distinct cell populations were detected using the SCTIME database.

### 2.9 Enzyme‐Linked Immunosorbent Assay (ELISA)

Bone marrow‐derived macrophages were isolated from the femurs and tibias of mice following the removal of surrounding muscle tissue. The bone marrow was then flushed out with PBS using a 26‐gauge needle. Cells were cultured in complete RPMI‐1640 medium supplemented with 10% fetal bovine serum (FBS) and 20 ng/mL macrophage colony‐stimulating factor (M‐CSF) for 7 days, with half the medium volume replaced every 3 days. SPP1‐ACP5^+^ macrophages were labeled with specific antibodies against SPP1 (ProteinTech, CL647‐22952) and ACP5 (Abcam, ab25568), followed by sorting via flow cytometry (BD Biosciences) based on laser scattering and fluorescence signals. Murine head and neck squamous cell carcinoma SCC7 cells were transfected with negative control (NC) siRNA and CD44‐targeting siRNA (Santa Cruz, sc‐35,534) for 48 h, followed by incubation with the supernatant of SPP1‐ACP5^+^ macrophages for an additional 24 h. The knockdown efficiency of CD44 was verified by western blotting using a specific antibody against CD44 (ProteinTech, 30854‐1‐AP). Subsequently, the cell culture supernatants were collected, and CXCL13 secretion was monitored using an ELISA kit (ProteinTech, KE10088) according to the manufacturer's instructions. In addition to that, culture supernatants of LILRB1‐transfected THP‐1 cells and SCC cells were collected and the concentration of SPP1 (ProteinTech, KE00375) and CXCL13 was measured using commercial ELISA kits following the manufacturer's instructions. All cell lines were authenticated by an expert before being used for experimentation and cell lines were free from mycoplasma contamination.

### 2.10 Western Blotting (WB)

Human THP‐1 cells were cultured in RPMI‐1640 with 10% FBS and 1% penicillin–streptomycin at 37°C/5% CO_2_. For LILRB1 overexpression, cells were transfected with LILRB1‐overexpressing plasmid or empty plasmid using Lipofectamine 3000 and transfection efficiency was verified by WB 48 h post‐transfection. Control and LILRB1‐overexpressing THP‐1 cells were differentiated into adherent macrophages with 100 ng/mL PMA for 48 h. Subsequently, control and LILRB1‐overexpressing THP‐1 cells were seeded into the upper chamber of transwell and induced to completely differentiate into macrophages for 48 h; SCC cells were seeded in the lower chamber at the same time. After continuous culture for 24 h, the protein of tumor cells in the lower chamber and the supernatant were extracted to detect the expression of CD44 (ProteinTech, 30854‐1‐AP) and CXCL13 (CST, 85679) of SCC cells.

### 2.11 Statistical Analysis

All statistical analyses were performed using online bioinformatics databases and the R software (v4.3.1), unless otherwise specified. The differential expression of LILRB1 in HNSCC tissues was determined using the student's *t*‐test. The log‐rank test was used to assess the significance of survival rate. Kruskal's test was employed to explore the association between LILRB1 expression and immune cell subtypes. Pearson's correlation coefficient was used to analyze the correlation between different genes. LR analysis was determined by the CellChat (v1.1.3) package. For multiple hypothesis testing, false discovery rate (FDR) correction was conducted using the Benjamini‐Hochberg (BH) method, with the FDR threshold set at 0.05 to filter out significant results. Two RNA‐seq normalization approaches including FPKM and TPM were chosen, as different bioinformatics databases employ distinct normalization strategies. DE threshold was set at FoldChange ≥ 2 or ≤ 0.5. The significance level was set at *p* < 0.05.

## 3 Results

### 3.1 High Level of LILRB1 Is Associated With a Good Prognosis of Male HNSCC Patients With High Tumor Mutation Burden

**FIGURE 1:**
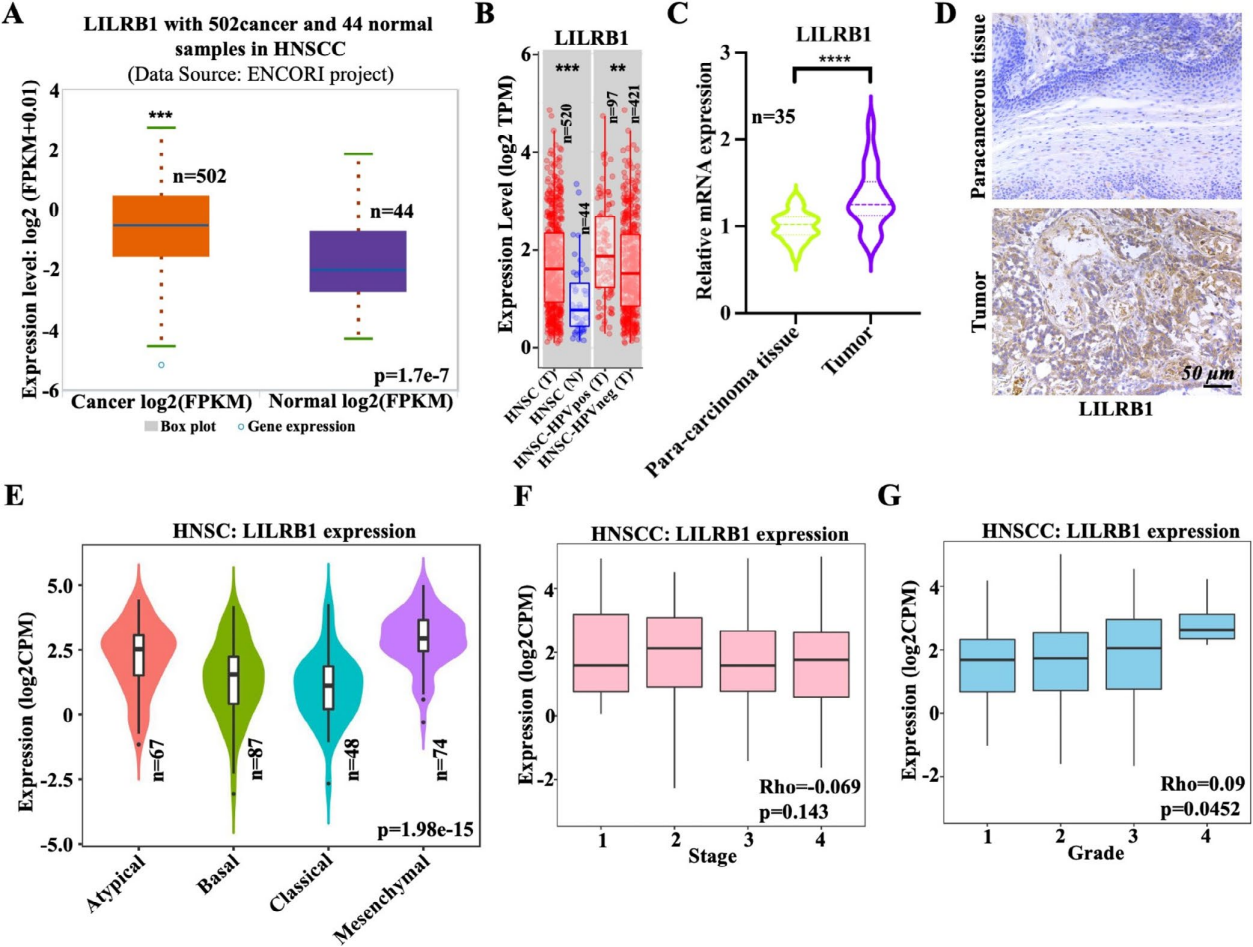
Differential expression of LILRB1 expression in head and neck squamous cell carcinoma (HNSCC). (A) Box plot from Encyclopedia of RNA Interactomes (ENCORI) database showing LILRB1 mRNA levels in cancer (*n* = 502) and normal samples (*n* = 44) of HNSCC. (B) Box plot from Tumor Immune Estimation Resource (TIMER) database showing LILRB1 mRNA levels in tumor (*n* = 520) and normal samples (*n* = 44) of HNSCC, as well as in HPV‐positive (*n* = 97) and HPV‐negative (*n* = 421) tumors. (C) LILRB1 mRNA levels in fresh tumor and paracancerous tissues of HNSCC as visualized by qRT‐PCR. (D) LILRB1 protein levels in fresh tumor and paracancerous tissues of HNSCC, as detected by immunohistochemical staining (IHC). Scale bar: 50 μm. (E) LILRB1 mRNA levels across different HNSCC subtypes including Atypical (*n* = 67), Basal (*n* = 87), Classical (*n* = 48), and Mesenchymal HNSCC (*n* = 74). (F, G) Box plot showing the relative expression of LILRB1 in normal subjects and HNSCC patients with different tumor stages (I, II, III, or IV) (F) or different tumor grades (1, 2, 3, 4) (G). **, *p* < 0.01; ***, *p* < 0.001.

**FIGURE 2:**
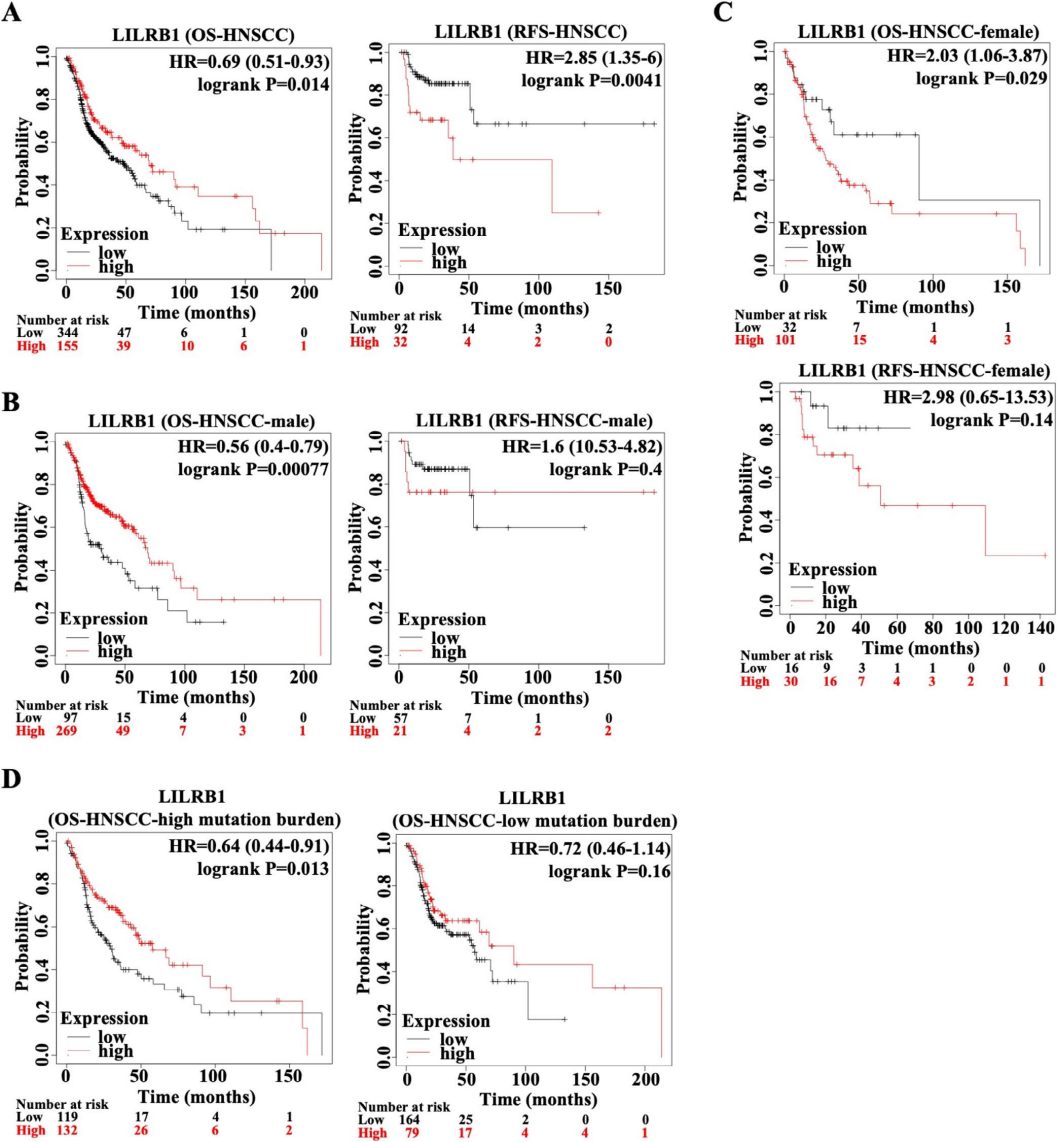
Clinical prognosis analysis of LILRB1 expression in HNSCC. (A) Left: Overall survival (OS) curve for HNSCC patients stratified by LILRB1 expression; Right: Relapse‐free survival (RFS) curve for HNSCC patients stratified by LILRB1 expression. (B, C) Left/Top: OS curve for male HNSCC patients stratified by LILRB1 expression (B) and female HNSCC patients stratified by LILRB1 expression (C); Right/below: RFS curve for male (B) or female (C) HNSCC patients stratified by LILRB1 expression. (D) OS curve for HNSCC patients with high/low tumor mutational burden (TMB) stratified by LILRB1 expression.

To evaluate the role of LILRB1 in HNSCC, LILRB1 transcription levels were analyzed using ENCORI and TIMER databases. Both datasets showed that LILRB1 was highly expressed in HNSCC tissues (particularly in human papillomavirus (HPV)‐positive HNSCC) relative to adjacent normal tissues (Figure 1A,B). This differential expression was further confirmed by qRT‐PCR using fresh HNSCC samples (Figure 1C). Immunohistochemical staining also showed upregulated LILRB1 protein levels in HNSCC tissues compared to paracancerous tissues (Figure 1D). Additionally, LILRB1 expression was highest in the mesenchymal HNSCC subtype compared to the other three subtypes (atypical, basal, and classical) (Figure 1E). However, LILRB1 expression did not differ among HNSCC patients at different stages (I‐IV) or with different tumor grades (I‐IV) (Figure 1F,G). Subsequently, the clinical significance of LILRB1 in HNSCC was assessed by analyzing OS and RFS in patients with differential LILRB1 expression. The results indicated that HNSCC patients in the low LILRB1 expression group (LILRB1^low^) had significantly poorer OS than those in the high LILRB1 expression group (LILRB1^high^) (Figure 2A). In contrast, LILRB1^high^ patients had shorter RFS than LILRB1^low^ patients (Figure 2A). Notably, LILRB1^high^ male HNSCC patients had a more favorable prognosis than LILRB1^low^ males (Figure 2B), in contrast to that of female HNSCC patients, who had a better prognosis in the LILRB1^low^ group than in the LILRB1^high^ group (Figure 2C). However, no such differences in RFS were observed between the LILRB1^high^ and LILRB1^low^ groups among male or female HNSCC patients (Figure 2B,C). Interestingly, a more favorable prognosis was observed in LILRB1^high^ HNSCC with high TMB compared to LILRB1^low^ patients with high TMB (Figure 2D). In contrast, LILRB1 expression was not associated with prognosis in HNSCC patients with low TMB (Figure 2D). Collectively, these findings suggest that high LILRB1 expression may predict a favorable prognosis in HNSCC, especially in male patients with high TMB.

### 3.2 LILRB1‐Coexpressed Genes Enrich in Multiple Signaling Pathways in HNSCC

**FIGURE 3:**
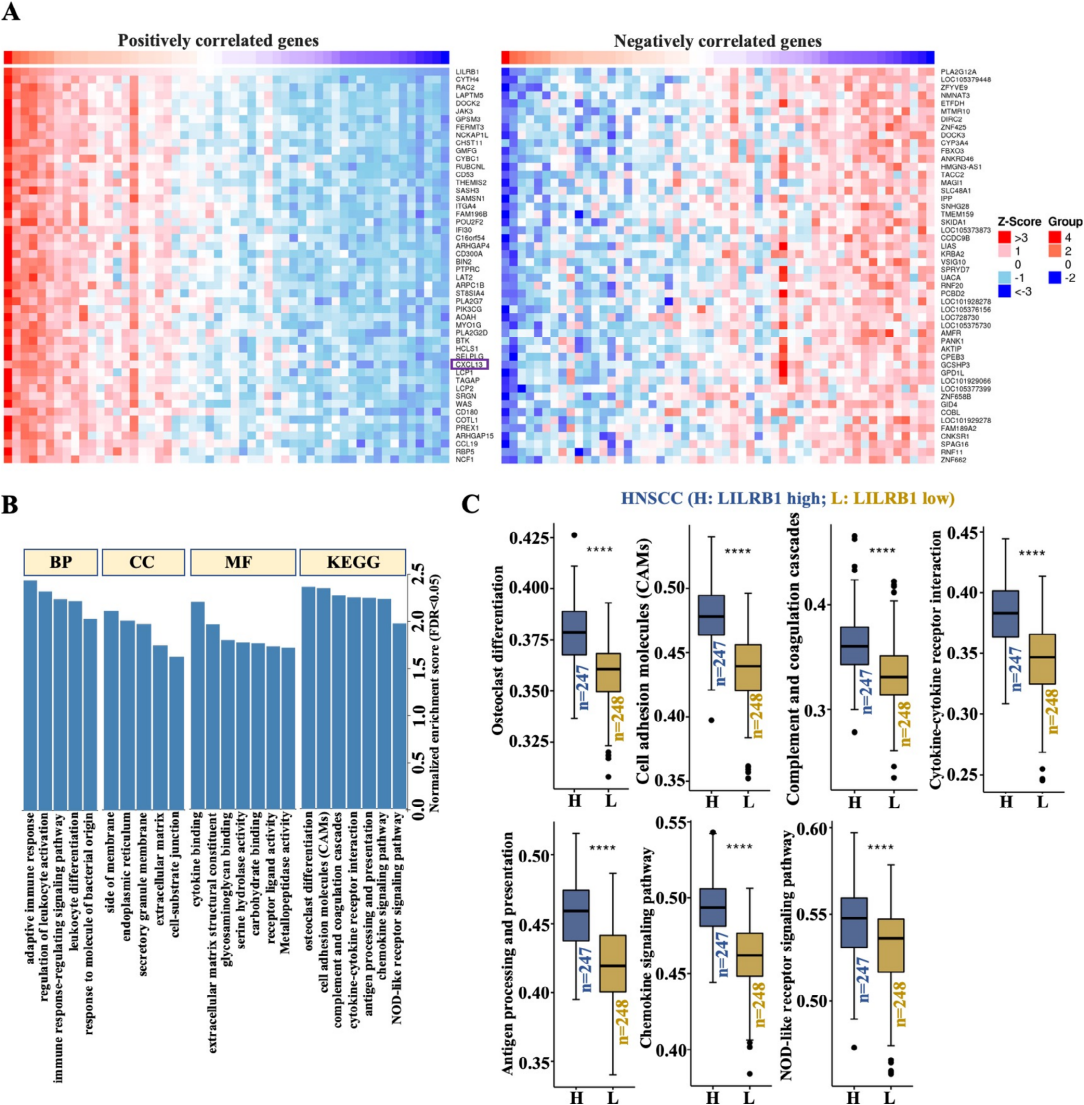
GO annotation/KEGG pathway enrichment analysis of LILRB1‐coexpressed genes in HNSCC. (A) Heatmap showing the top 50 genes positively and negatively correlated with LILRB1 in HNSCC. Red color indicated a positive correlation, and green color indicated a negative correlation. (B) Gene set enrichment analysis (GSEA) was performed to assess GO annotations and KEGG pathway enrichment of LILRB1‐coexpressed genes in HNSCC. BP, biological processes; CC, cellular components; MF, molecular functions. False discovery rate (FDR) from GSEA was 0. (C) Positively enriched pathways associated with differential LILRB1 expression (*n* = 247/248) as confirmed via the single‐sample GSEA (ssGSEA) method using the Comprehensive Analysis on Multi‐Omics of Immunotherapy in Pan‐cancer (CAMOIP) database. ****, *p* < 0.0001.

Using the Linkedomics database, LILRB1‐coexpressed genes were identified, and the top 50 positively and negatively correlated genes with LILRB1 are presented as heatmaps (Figure 3A,B). The top‐ranked positively related genes included CYTH4, RAC2, LAPTM5, and the chemokine CXCL13, while the top‐ranked negatively correlated genes included LOC105379448, ZFYVE9, NMNAT3, and ETFDH (Figure 3A). GO enrichment analysis revealed that LILRB1‐coexpressed genes were involved in biological processes (BP) including adaptive immune response, regulation of leukocyte activation, immune response‐regulating signaling pathways, leukocyte differentiation, and response to molecules of bacterial origin (Figure 3B). For cellular component (CC) analysis, these genes were primarily localized to the side of the membrane, endoplasmic reticulum, secretory granule membrane, extracellular matrix, and cell‐substrate junction. Molecular function (MF) results showed that cytokine binding, extracellular matrix structural constituents, glycosaminoglycan binding, serine hydrolase activity, carbohydrate binding, receptor ligand activity, and metallopeptidase activity were significantly associated with LILRB1‐related genes (Figure 3B). KEGG pathway enrichment analysis indicated that the pathways enriched by LILRB1‐coexpressed genes included osteoclast differentiation, cell adhesion molecules (CAMs), complement and coagulation cascades, cytokine‐cytokine receptor interaction, antigen processing and presentation, chemokine signaling pathway, and NOD‐like receptor signaling pathway (Figure 3B). These enriched pathways were further validated using single‐sample gene set enrichment analysis (ssGSEA). The results showed that LILRB1^high^ HNSCC patients (*n* = 247) exhibited higher activity in the aforementioned pathways compared to LILRB1^low^ HNSCC patients (*n* = 248) (Figure 3C). Combined with the GO/KEGG enrichment analysis, LILRB1‐coexpressed genes are involved in multiple signaling pathways in HNSCC.

### 3.3 LILRB1 Is Positively Associated With Immune Effector Cell Infiltration in HNSCC

**FIGURE 4:**
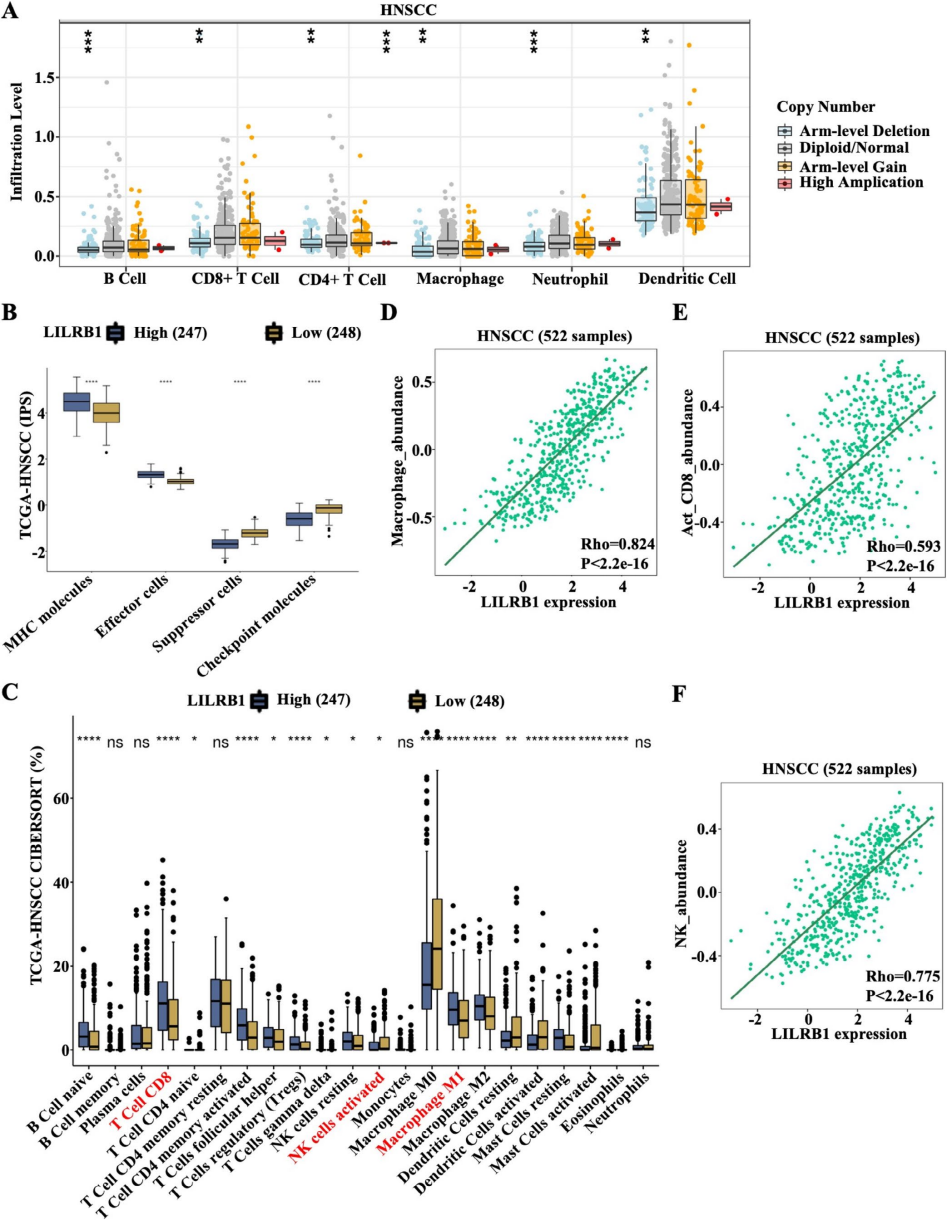
Correlation between LILRB1 expression and immune infiltration in HNSCC. (A) Association between LILRB1 copy number variation (CNV) and the infiltration level of B cells, CD8^+^ T cells, CD4^+^ T cells, Macrophages, Neutrophils and dendritic cells (DCs) in HNSCC. (B) Abundances of major histocompatibility complex (MHC) molecules, Effector cells, Suppressor cells, and Checkpoint molecules in low and high LILRB1 expression groups. (C) Distribution of 22 immune cell subtypes in LILRB1^low^ and LILRB1^high^ HNSCC groups. (D–F) Association between LILRB1 expression and macrophage abundance (D), CD8^+^ T cell abundance (E), and natural killer (NK) cell abundance in HNSCC (F), as analyzed using the TIMER database. *, *p* < 0.05; **, *p* < 0.01; ***, *p* < 0.001; ****, *p* < 0.0001.

Subsequently, the association between LILRB1 expression and immune cell infiltration in HNSCC was explored using the TIMER database. As shown in Figure 4A, CNV of LILRB1 was associated with the infiltration levels of B cells, CD8^+^ T cells, CD4^+^ T cells, macrophages, neutrophils, and DCs (Figure 4A). Using the computing method of IPS (TCGA‐HNSCC), LILRB1^high^ HNSCC patients (*n* = 247) exhibited higher levels of MHC molecules and effector cells, as well as lower levels of suppressor cells and immune checkpoint molecules compared to LILRB1^low^ patients (*n* = 248) (Figure 4B). Using the computing method of CIBERSORT (TCGA‐HNSCC), the differences in 22 types of TIICs between LILRB1^high^ and LILRB1^low^ HNSCC were compared. The data showed that LILRB1^high^ patients harbored higher proportions of naive B cells, CD8^+^ T cells, activated CD4^+^ memory T cells, follicular helper T cells, regulatory T cells (Tregs), resting DCs, and both M1 and M2 macrophages (Figure 4C). Additionally, a significant positive association between LILRB1 and the abundance of immune cells, including macrophages, activated CD8^+^ T cells, NK cells, activated CD4^+^ T cells, and activated B cells, was identified in HNSCC using the TISIDB database (Figure 4D,F and Figure S2B,C). However, more immunosuppressive cell types, such as MDSCs and Tregs, were also enriched in LILRB1^high^ HNSCC tissues (Figure 4C and Figure S2A). Therefore, LILRB1 exerts a dual role in the TME during the progression of HNSCC. Balancing these opposing effects represents an urgent challenge to address.

### 3.4 Prognostic Effect of LILRB1 Depends on Immune Effector Cell Infiltration in HNSCC

**FIGURE 5:**
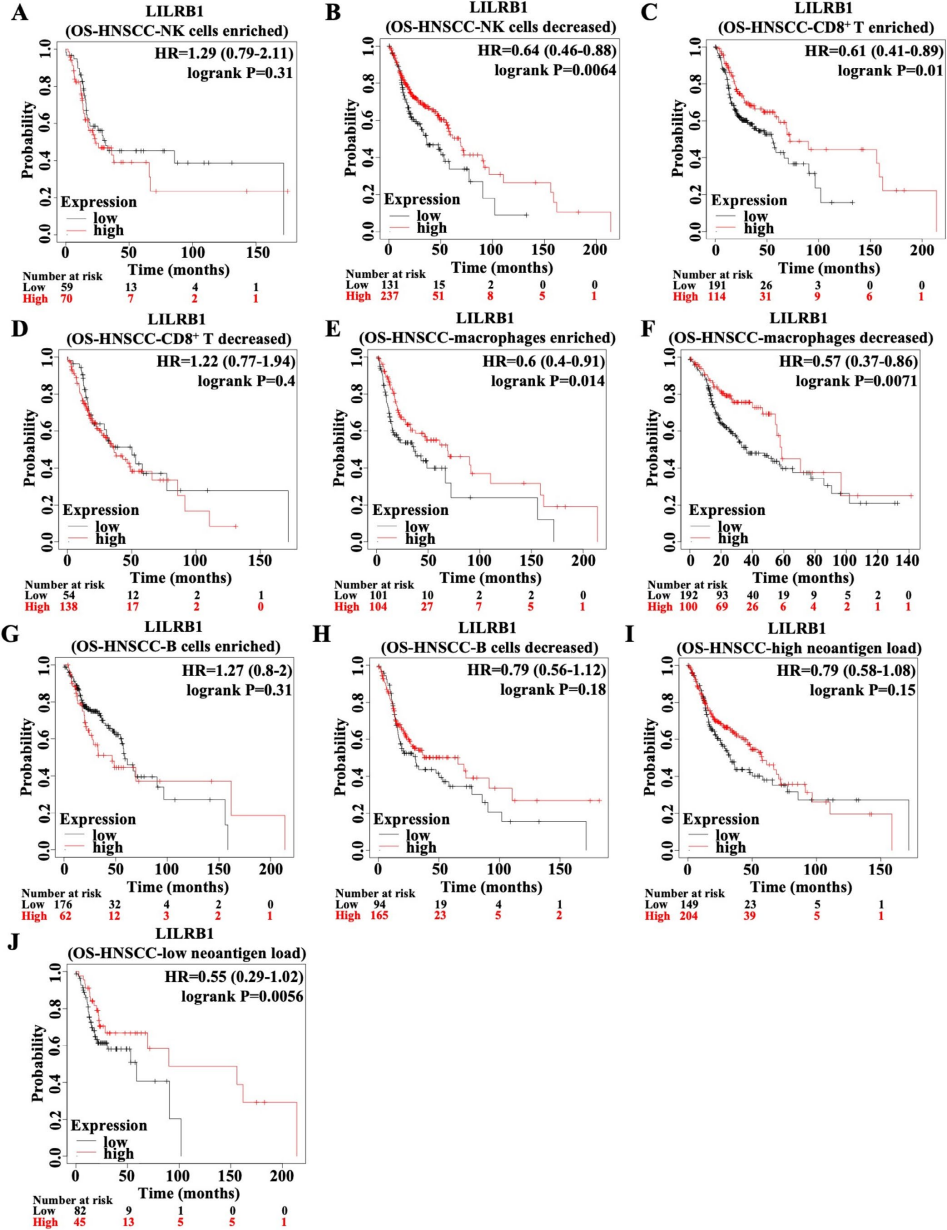
Clinical prognostic analysis of LILRB1 expression in HNSCC stratified by immune cell infiltration. (A, B) OS curve for HNSCC with high NK cell infiltration (A) and low NK cell infiltration (B), stratified by LILRB1 expression. (C, D) OS curve for HNSCC with high CD8+ T cell infiltration (C) and low CD8+ T cell infiltration (D), stratified by LILRB1 expression. (E, F) OS curve for HNSCC with high macrophage infiltration (E) and low macrophage infiltration (F), stratified by LILRB1 expression. (G, H) OS curve for HNSCC with high B cell infiltration (G) and low B cell infiltration (H), stratified by LILRB1 expression. (I, J) OS curve for HNSCC with high neoantigen load (I) and low neoantigen load (J), stratified by LILRB1 expression.

KM Plotter was used to determine whether LILRB1‐mediated immune cell infiltration influences the prognosis of HNSCC patients. The data indicated no significant difference in OS between LILRB1^high^ and LILRB1^low^ HNSCC with high NK cell infiltration. However, LILRB1^high^ patients with low NK cell infiltration presented a more favorable prognosis compared to LILRB1^low^ patients, suggesting that the prognostic effect of LILRB1 in HNSCC is dependent on NK cell abundance (Figure 5A,B). In contrast, LILRB1^high^ patients harbored a better outcome only in the subgroup with high CD8^+^ T cell infiltration, whereas no such advantage was observed in patients with low CD8^+^ T cell infiltration (Figure 5C,D). Additionally, B cell and macrophage abundance did not modulate the prognostic role of LILRB1 in HNSCC: high LILRB1 expression was associated with more favorable OS in both patients with high and low macrophage infiltration (Figure 5E–H). In addition, LILRB1^high^ patients had a better outcome only in HNSCC patients with low neoantigen load compared to LILRB1^low^ patients, with no significant difference observed in those with high neoantigen load (Figure 5I,J). Therefore, the prognostic effects of LILRB1 in HNSCC are primarily dependent on CD8+ T cell abundance, especially in patients with low neoantigen load.

### 3.5 High LILRB1 Expression Harbors a High Immune Score in HNSCC and Predicts Favorable Responses to Immunotherapy

**FIGURE 6:**
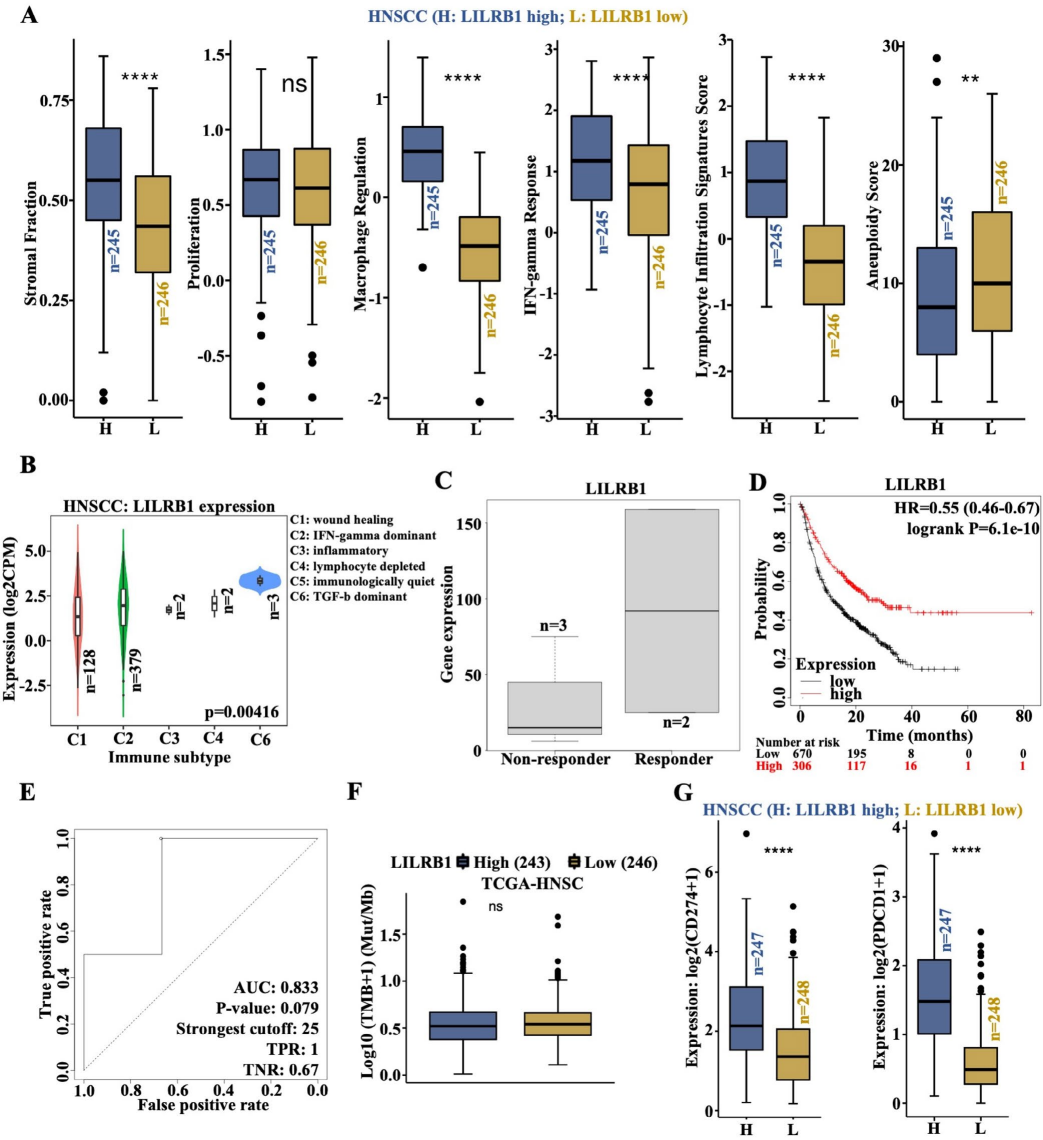
Immune‐related scores and immunotherapeutic response analysis in HNSCC patients stratified by LILRB1 expression. (A) Immune‐related indexes scores including Stromal Fraction, Proliferation, Macrophage Regulation, IFN‐gamma Response, Lymphocyte Infiltration Signatures Score, and Aneuploidy Score in LILRB1^high^ (*n* = 245) and LILRB1^low^ (*n* = 246) HNSCC patients. (B) LILRB1 expression pattern across different HNSCC immune subtypes (C1–C6, *n* = 128/379/2/2/3). (C) Differences in LILRB1 expression between non‐responder (*n* = 3) and responder (*n* = 2) among HNSCC patients who received immunotherapy. (D) OS curve for patients with various tumor types (including five HNSCC patients who received immunotherapy), stratified by LILRB1 expression. (E) Area under the curve (AUC) of LILRB1 as a predictor for HNSCC patients who received immunotherapy. (F) Analysis of differences in TMB between LILRB1^high^ (*n* = 243) and LILRB1^low^ (*n* = 246) HNSCC patients. (G) Expression analysis of PD‐L1 (CD274) and PD1 (PDCD1) in LILRB1^high^ (*n* = 247) and LILRB1^low^ (*n* = 248) HNSCC patients. **, *p* < 0.01; ****, *p* < 0.0001.

The immune score of HNSCC patients with differential LILRB1 expression was analyzed using the CAMOIP database. No significant association was observed between LILRB1 expression and immune cell proliferation in HNSCC patients (Figure 6A). In addition, multiple immune score indices, including stromal fraction, macrophage regulation, IFN‐gamma response, and lymphocyte infiltration signature score, were significantly higher in LILRB1^high^ HNSCC patients than in LILRB1^low^ patients (Figure 6A). In contrast, the aneuploidy Score, another immune‐related index, showed a significant negative association with LILRB1 expression in HNSCC patients (Figure 6A). Moreover, LILRB1 expression was highest in the TGF‐β‐dominant immune subtype of HNSCC, compared to the other five immune subtypes, including wound healing, IFN‐gamma dominant, inflammatory, lymphocyte depleted, and immunologically quiet, which indicated that LILRB1 plays a critical role in shaping the TME of HNSCC (Figure 6B). Importantly, LILRB1 was highly expressed in responders compared to non‐responders among HNSCC patients who received immunotherapy (Figure 6C). Among five HNSCC patients who received immunotherapy, a better prognosis was observed in LILRB1^high^ patients than in LILRB1^low^ patients (Figure 6D). In this small cohort (*n* = 5), LILRB1 expression showed a tend toward predictive value for immunotherapy response (Area Under the Curve: AUC = 0.833, *p* = 0.079) (Figure 6E). Additionally, no significant association was observed between LILRB1 expression and TMB in HNSCC patients (Figure 6F). When analyzing the correlation between LILRB1 and PD‐1/PD‐L1 signaling pathways, PD‐L1 (CD274) and PD‐1 (PDCD1) expression levels were significantly higher in LILRB1^high^ HNSCC patients than in LILRB1^low^ patients (Figure 6G). Taken together, high LILRB1 expression correlates with a high immune score in HNSCC and predicts a favorable prognosis in patients receiving immunotherapy. Possibly, LILRB1 may serve as a potential biomarker for evaluating the efficacy of PD‐1/PD‐L1‐targeted immunotherapy in HNSCC.

### 3.6 LILRB1 Regulates the Interaction Between Macrophage and Cancer Cells Through SPP1‐CD44 Axis

**FIGURE 7:**
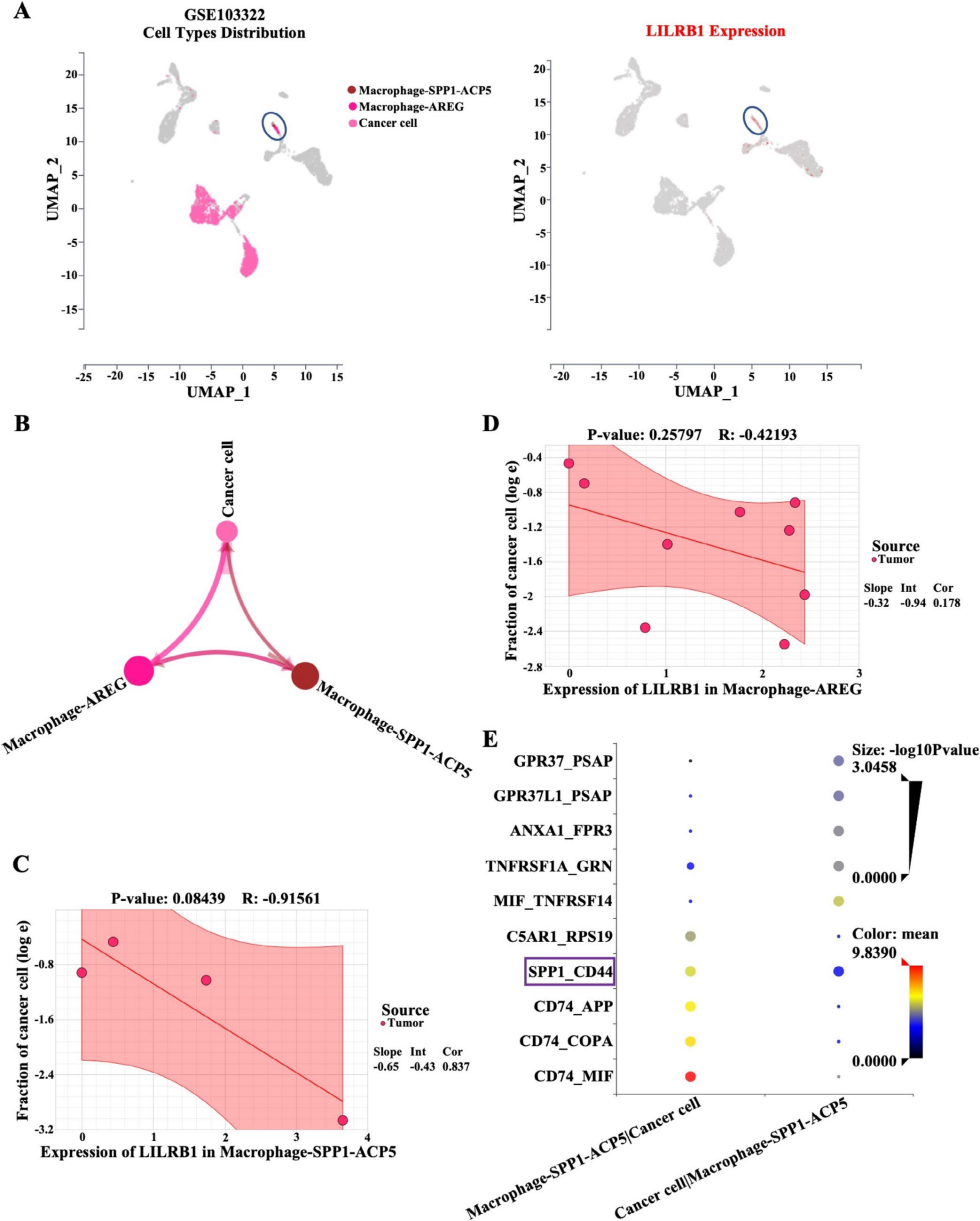
Cell‐to‐cell communications analysis in HNSCC patients stratified by LILRB1 expression. (A) UMAP plot showing the distribution of selected cell types (SPP1‐ACP5^+^ Macrophage, macrophage‐AREG, and cancer cell) integrated from single‐cell RNA sequencing data retrieved from Gene Expression Omnibus (GSE103322) (left); Single‐cell transcriptomic expression of LILRB1 across different cell types retrieved from GSE103322 (right). (B) Circle plots show the overview of the number of cell–cell interactions among Macrophage‐SPP1‐ACP5, macrophage‐AREG, and cancer cell based on single cell RNA‐seq data. (C) Correlation analysis between LILRB1 expression in Macrophage‐SPP1‐ACP5 and cancer cell composition. (D) Correlation analysis between LILRB1 expression in Macrophage‐AREG and cancer cell composition. (E) Dot plot showing Ligand‐receptor (LR) pair analysis between Macrophage‐SPP1‐ACP5 and cancer cells (left), and between cancer cells and Macrophage‐SPP1‐ACP5 (right). X‐axis: Signal direction from macrophage‐SPP1‐ACP5 to Cancer cell, and from Cancer cell to macrophage‐SPP1‐ACP5. Y‐axis: Specific LR pairs are listed. The size of the points indicates statistical significance (larger point values means smaller *p*‐values, and more significant interactions). Point color represents the interaction strength, with a color gradient from blue to red, where red indicates higher intensity.

A previously published single‐cell dataset (GSE103322) was utilized to characterize the effect of LILRB1 in mediating crosstalk between macrophages and cancer cells in HNSCC. The data demonstrated that LILRB1 was predominantly expressed in SPP1‐ACP5^+^ and AREG^+^ macrophage subsets (Figure 7A). Circle plots showed extensive crosstalk between SPP1‐ACP5^+^ macrophages/AREG^+^ macrophages and cancer cells within HNSCC tumors (Figure 7B). Correlation analysis between LILRB1 expression and cell composition showed that HNSCC patients with high LILRB1 in SPP1‐ACP5^+^ macrophages had a lower fraction of cancer cells than those with low LILRB1 expression. Although this difference did not reach statistical significance (*p* = 0.084), a trend toward a negative correlation was observed (Figure 7C). However, no obvious change in cancer cell fraction was observed in HNSCC patients with high LILRB1 expression in AREG^+^ macrophages (Figure 7D). These findings indicate that LILRB1 expressed in SPP1‐ACP5^+^ macrophages may regulate the cell–cell interactions between macrophages and cancer cells. Subsequent ligand receptor (LR) analysis showed that the CD74‐MIF, CD74‐COPA, CD74‐APP, and SPP1‐CD44 signaling axes mediated the interaction between SPP1‐ACP5^+^ macrophages and cancer cells (Figure 7E). Combining the single‐cell dataset (GSE103322), the specific expression of SPP1 and ACP5 was confirmed in SPP1‐ACP5^+^ macrophages, whereas CD44 was mainly expressed in cancer cells (Figure S3A,C). Additionally, LILRB1^high^ HNSCC patients harbored higher levels of SPP1 and ACP5, while no significant difference in CD44 expression was observed between LILRB1^high^ and LILRB1^low^ HNSCC patients (Figure S3D). Taken together, these findings demonstrate that LILRB1 in SPP1‐ACP5+ macrophages mediates crosstalk between this macrophage subset and cancer cells, likely through the SPP1‐CD44 signaling axis.

### 3.7 Macrophage LILRB1 Regulates CD8 ^+^ T Cell Abundance Through CXCL13‐CXCR5 Axis

**FIGURE 8:**
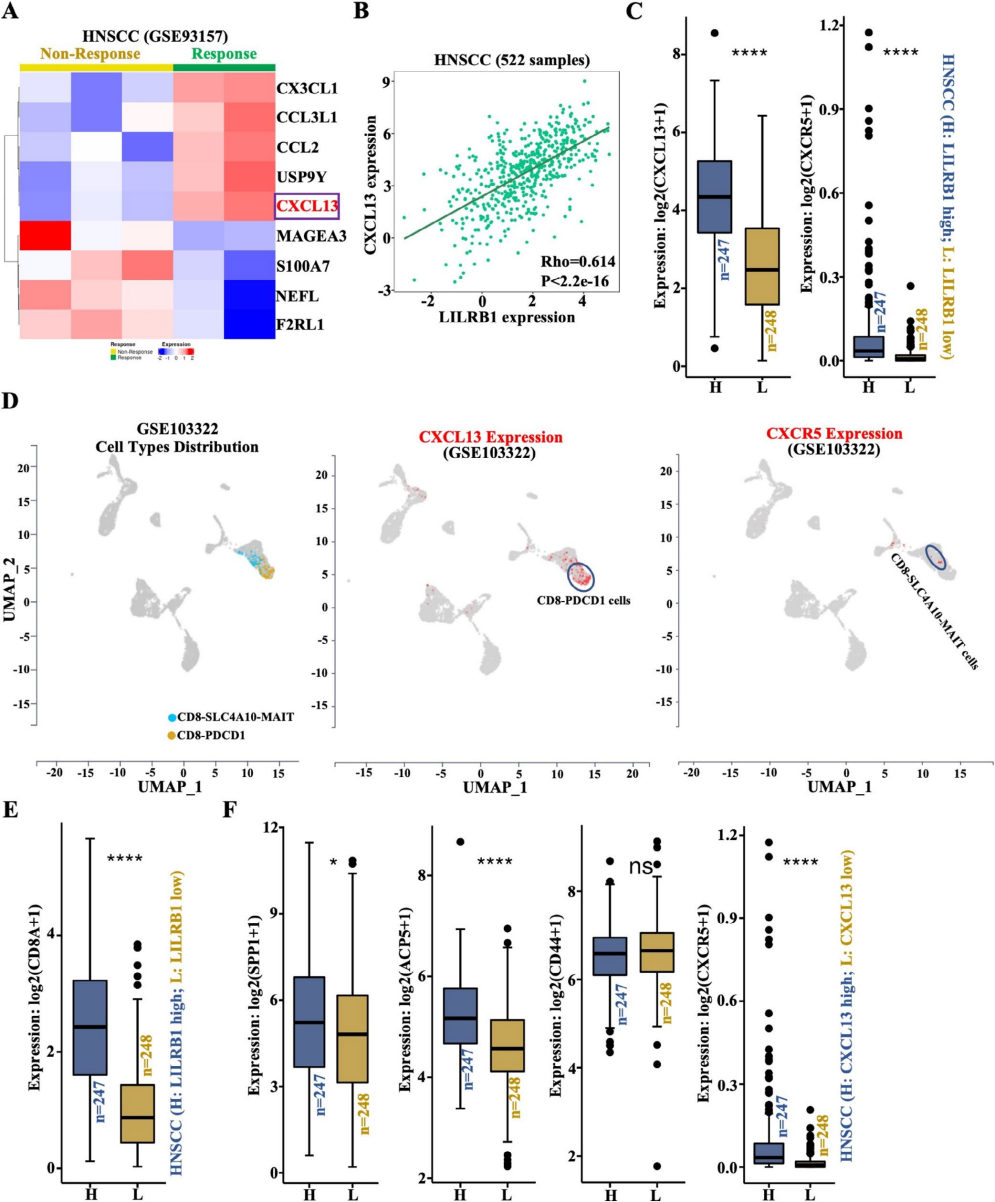
Ligand‐receptor (LR) network analysis of crosstalk among DCs, HPC‐like cells, malignant cells and macrophage. (A) Heatmap showing DEGs in HNSCC patients who received immunotherapy. (B) Association between LILRB1 and CXCL13 expression in HNSCC (*n* = 522). (C) Histogram visually displaying differential expression of CXCL13 and CXCR5 between LILRB1^high^ (*n* = 247) and LILRB1^low^ (*n* = 248) HNSCC patients. (D) The left UMAP plot showing the distribution of cell subsets (CD8‐SLC4A10‐MAIT and CD8‐PDCD1) integrated from single‐cell data that were retrieved from GSE103322. Two UMAP plots showing expression distribution of CXCL13 in CD8‐PDCD1 and CXCR5 in CD8‐SLC4A10‐MAIT (right). (E) Histogram visually displaying differential expression of CD8A between LILRB1^high^ (*n* = 247) and LILRB1^low^ (*n* = 248) HNSCC patients. (F) Histogram visually displaying differential expression of SPP1, ACP5, CD44, and CXCR5 between CXCL13 high (*n* = 247) and CXCL13 low (*n* = 248) HNSCC patients. ****, *p* < 0.0001.

Among HNSCC patients who responded to immunotherapy, the expression levels of *CX3CL1*, *CCL3L1*, *CCL2*, *USP9Y*, and *CXCL13* were notably upregulated, while *MAGEA3*, *S100A7*, *NEFL*, and *F2RL1* were downregulated compared to non‐responders (Figure 8A). Consistent with our earlier findings in Figure 3A, *CXCL13* was a significantly positively co‐expressed gene of LILRB1 in HNSCC (Figure 3A). Our data further demonstrated a significant positive association between *LILRB1* and *CXCL13* expression in HNSCC (Figure 8B). A significant positive correlation was also observed between *LILRB1* and *CXCR5*, the unique receptor for CXCL13, in HNSCC (Figure S3E). LILRB1^high^ HNSCC patients exhibited significantly higher levels of *CXCL13* and *CXCR5* than LILRB1^low^ patients (Figure 8C). Combined with the single‐cell dataset (GSE103322), *CXCL13* was primarily expressed in PDCD1 (PD‐1)^+^ CD8^+^ T cells, while *CXCR5* was predominantly located in SLC4A10‐MAIT^+^ CD8^+^ T cells (Figure 8D). A previous study confirmed that CXCL13 recruits CXCR5^+^‐CD8^+^ T lymphocytes to tertiary lymphoid structures (TLSs), which in turn promotes CXCL13 secretion to recruit additional CXCR5^+^ immune cells and facilitate cancer cell targeting [22]. Our data also showed a higher expression of CD8A in LILRB1^high^ HNSCC patients than those with low LILRB1 expression (Figure 8E). Therefore, macrophage‐derived LILRB1 may affect CD8^+^ T cell enrichment via the CXCL13‐CXCR5 axis. Our study further indicated a significant positive correlation between the expression of SPP1/ACP5/CXCR5 and CXCL13 expression in HNSCC (Figure 8F). Importantly, a significant positive correlation was also found between LILRB1 and a six‐gene signature (SPP1/ACP5/CD44/CXCL13/CXCR5/CD8A) in HNSCC (Figure S3F). After the coculture of macrophages with CD44‐deficient SCC7 cancer cells, CXCL13 secretion by cancer cells was notably restrained (Figure S4A,B). Once LILRB1 was overexpressed in macrophages (THP‐1 cells), more SPP1 expression was observed accompanied by the overexpression of LILRB1 (Figure S4C,D). To further confirm the downstream signaling pathway, control and LILRB1 overexpressing THP‐1 cells were seeded into the upper chamber of transwell and induced to completely differentiate into macrophages for 48 h; SCC cells were seeded in the lower chamber at the same time. After continuous culture for 24 h, the protein of tumor cells in the lower chamber and the supernatant were extracted to detect the expression of CD44/CXCL13 and CXCL13 secretion of SCC cells. The data proved that co‐culture of LILRB1‐overexpressing macrophages with SCC cells significantly promoted protein expression of CD44 and CXCL13, along with the increase of CXCL13 secretion (Figure S4E,G). Given that CD44 is also expressed on CD8^+^ T cells (Figure S3C and Figure 8D), we hypothesize that macrophage‐derived LILRB1 may mediate the interaction between SPP1‐ACP5^+^ macrophages and CD8^+^ T cells via the SPP1‐CD44 axis. This interaction subsequently promotes CXCL13 secretion by cancer cells or T cells, which recruits additional CXCR5^+^‐CD8^+^ T cells and induces CD8^+^ T cell immune activation to lyse cancer cells.

## 4 Discussion

HNSCC exhibits significant genetic heterogeneity, which poses challenges to the classification of patients and the development of personalized targeted therapies [23]. Traditional treatment strategies such as surgery, radiotherapy, or chemotherapy for HNSCC are often inadequate and prone to inducing treatment resistance. Accumulating evidence suggests that immune checkpoints blockade (ICB) can restore the cytotoxic function of immune cells, prompting the development of treatments aimed at reactivating anti‐tumor immune response within the TME of HNSCC [24, 25]. However, current PD‐1/PD‐L1‐targeted ICB therapies for HNSCC, such as pembrolizumab, nivolumab, and durvalumab, still suffer from limited response rates and suboptimal safety profiles [26]. In this context, high LILRB1 expression correlates with favorable prognosis in HNSCC and may activate crosstalk between SPP1/ACP5^+^ macrophages and cytotoxic T cells, thereby shaping the TME via the CXCL13/CXCR5 signaling axis. Taken together, combining LILRB1‐targeted therapy with other ICB agents may elicit a synergistic and robust anti‐tumor immune response in HNSCC.

LILRB1 is also expressed on the surface of solid tumor cells. Accumulating evidence has demonstrated that LILRB1 is highly expressed in gastric cancer tissues and cell lines, with its expression correlating with the degree of gastric cancer differentiation [27]. In hepatocellular carcinoma, LILRB1 is highly expressed in immune cells but weakly expressed in cancer cells [28, 29]. In HNSCC, high levels of LILRB1 were observed in tumor tissues, particularly in HPV‐positive tumor tissues, compared to normal or para‐carcinoma tissues of HNSCC. In gastric cancer, high LILRB1 expression is associated with advanced tumor stage, increased recurrence risk, and poor overall survival [30]. In contrast, LILRB1 expression showed no significant correlation with tumor stage or grade in HNSCC. Although the primary function of immune checkpoint molecules is to mediate immune escape, they are also widely expressed on tumor cells, where they play crucial roles in sustaining malignant behaviors such as epithelial‐mesenchymal transition (EMT) [31]. LILRB1 expression was highest in the mesenchymal HNSCC subtype, suggesting that LILRB1 may regulate EMT progression or tumor metastasis. In glioma patients, elevated LILRB1 expression predicts poor prognosis, and LILRB1 combined with TMB and microsatellite instability may serve as a promising predictor of immunotherapy response [32]. Despite high LILRB1 expression in HNSCC, patients with high LILRB1 expression had a more favorable prognosis compared to those with low LILRB1 expression. However, the predictive role of LILRB1 was heterogeneous across male and female HNSCC patients, as well as between patients with high and low TMB. This heterogeneity may be attributed to cell type‐specific functions of LILRB1, as well as differences in patient sex and TMB levels. Actually, recent studies have showed an association between sex hormone receptors and the pathogenesis/prognosis in patients with HNSCC [33]. Presumably, the opposite survival trends between male and female patients can be attributed to the differential effects of hormones. Additionally, LILRB1 status did not affect the overall survival of male HNSCC patients with low TMB, which was exactly the opposite of that in male patients with high TMB. However, HNSCC patients with high LILRB1 levels showed a better prognosis in male patients with either high or low neoantigen load. The data indicated that the improved prognosis in male HNSCC patients with high LILRB1 expression is related to TMB difference, rather than neoantigen load (Figure S5A,B,E,F). On the contrary, in female HNSCC patients, both the TMB status and the neoantigen load significantly affected the improvement of prognosis in those with low LILRB1. The data indicate that the improved prognosis in female HNSCC patients with low LILRB1 is related to the differences in TMB and neoantigen load (Figure S5C,D,G,H). In conclusion, the opposite survival trends between male and female patients may be closely related to the interaction between hormone regulation and immune microenvironment.

In esophageal adenocarcinoma, the activation of chemokine‐receptor axes, accompanied by LILRB1 upregulation, is a key driver of immune escape, which in turn promotes cancer cell proliferation and metastasis [34]. Herein, LILRB1 and its co‐expressed genes were primarily enriched in several signaling pathways, including cell adhesion molecules (CAMs), cytokine‐cytokine receptor interactions, and chemokine signaling pathways. CXCL13, a gene positively co‐expressed with LILRB1, was also identified using the LinkedOmics online database. Thus, LILRB1 is closely associated with the chemokine signaling pathway in HNSCC. The MHC1/LILRB1 axis has been proposed as an innate immune checkpoint for cancer immunotherapy [35]. LILRB1 deficiency inhibits multiple myeloma (MM) progression in vivo by promoting ferroptosis in MM cells, implicating LILRB1 as a promising therapeutic target for MM patients [36]. In ovarian cancer, LILRB1 expression is associated with increased M2 macrophage infiltration, impaired dendritic cell activation, and CD8^+^ T cell dysfunction, all of which are indicative of an immunosuppressive phenotype [37]. However, high expression of LILRB family expression including LILRB1 correlates with favorable prognosis in liver cancer patients and is positively associated with increased immune cell infiltration, consistent with an immunostimulatory tumor phenotype [38]. Therefore, LILRB1 exerts a dual immunological role during the progression of multiple cancer types. In this study, HNSCC patients with high LILRB1 expression harbored a higher abundance of effector cells and a lower abundance of suppressor cells than those with low LILRB1 expression. This is evidenced by increased infiltration of CD8^+^ T cells and M1 macrophages in LILRB1^high^ HNSCC patients. Actually, co‐culture of NK cells with prostate cancer cells induces the expression of inhibitory receptors (ILT2/LILRB1), which in turn impairs NK cell anti‐tumor activity [39]. Our findings also proved that high LILRB1 expression predicted a good prognosis in HNSCC, which is associated with NK cell function and dependent on CD8^+^ T cell enrichment. The MHC‐I/LILRB1 signaling axis has been reported to be an important regulator of the effector function of innate immune cells, in part by regulating macrophage phagocytosis [40]. Although LILRB1 expression is positively associated with macrophage infiltration, macrophage abundance does not influence the predictive value of LILRB1 in HNSCC. Thus, we suspect that LILRB1 influences HNSCC prognosis, primarily by affecting the functional activity of the CD8^+^ T cells. Immunogenic cell death score‐related genes including DNASE1L3, KLRB1, and LILRB1 have been shown to potentially predict outcomes and therapeutic responses in liver cancer patients [41]. In tumors with low TMB, a higher aneuploidy score correlates with poor prognosis following immunotherapy [42]. In this study, LILRB1^high^ HNSCC patients exhibited higher immune scores and lower aneuploidy scores, which suggests favorable prognosis following immunotherapy. Indeed, LILRB1 expression is enhanced in HNSCC patients who responded to immunotherapy, and LILRB1^high^ patients had a longer overall survival than LILRB1^low^ patients. Therefore, evaluation of LILRB1 expression may assist clinicians in designing personalized treatment strategies for HNSCC patients.

LILRB1 is expressed in a variety of immune cells, including macrophages and specific subsets of cytotoxic lymphocytes [43]. In HNSCC, LILRB1 is predominantly expressed in macrophages. Disrupting the interaction between SPP1^+^ macrophages and FAP^+^ fibroblasts has been proposed as a potential therapeutic strategy to improve immunotherapy efficacy in colorectal cancer [44]. Tartrate‐resistant acid phosphatase (TRAP/ACP5), a metalloenzyme mainly expressed in activated osteoclasts and macrophages, is associated with cancer progression and increased tumor aggressiveness [45]. The SPP1‐CD44 ligand‐receptor pair may influence the function of regulatory T cells (Tregs) within the TME and modulate responses to HNSCC immunotherapy [46]. In HNSCC, LILRB1 co‐localizes with SPP1 and ACP5 in macrophages; furthermore, LILRB1 expression in SPP1‐ACP5^+^ macrophages is negatively correlated with cancer cell fraction. The crosstalk between SPP1‐ACP5^+^ macrophages and cancer cells is likely mediated by the SPP1‐CD44 signaling axis. It is plausible that LILRB1 expression reverses the pro‐tumorigenic role of SPP1 and ACP5 in cancer progression.

CXCL13 combined with other genes can be used to identify high‐risk HNSCC patients with poor prognosis [47]. In HNSCC, CXCL13 expression is also elevated in immunotherapy responders and positively associated with LILRB1 expression. CXCR5, a G‐protein coupled receptor, is the specific receptor of CXCL13, and the CXCL13‐CXCR5 axis plays a critical role in regulating inflammatory diseases and cancer progression [48]. HPV‐positive HNSCC patients typically have a better prognosis, attributed to increased B lymphocyte infiltration in the TME driven by the CXCL13‐CXCR5 axis [49]. Our data also showed a positive association between LILRB1 and CXCR5 expression in HNSCC. The presence of CXCR5^+^‐CD8^+^ follicular cytotoxic T cells is strongly associated with CD8^+^ effector function, which modulates anti‐tumor activity by regulating CD19^+^‐CD38^+^ B cells and TLSs [50]. In gastric cancer, high CXCL13 expression is associated with a more favorable prognosis, as it promotes increased infiltration of CXCR5^+^‐CD8^+^ T cells into TLSs [51]. High‐grade serous ovarian cancer with high CXCL13 expression exhibits increased infiltration and expansion of CXCR5^+^‐CD8^+^ T cells, facilitating the maintenance of CD8^+^ T cells' cytotoxicity [52]. We found that CXCL13 and CXCR5 were expressed in PDCD1‐CD8^+^ and SLC4A10‐MAIT‐CD8^+^ T cells, respectively. The data indicated that LILRB1^high^ HNSCC patients had more activated CD8^+^ T cells, indicating that the LILRB1‐coexpressed gene CXCL13 may promote the expansion and activation of CD8^+^ T cells by binding to CXCR5. Upon inhibition of the PD‐1/PD‐L1 signaling pathway, CXCL13 recruits CXCR5^+^‐CD8^+^ T lymphocytes to TLSs; these cells then secrete additional CXCL13 to recruit more CXCR5^+^ immune cells and mediate cancer cell lysis [22]. Assessing CXCL13 expression can effectively identify both precursor and terminally differentiated tumor‐reactive CD8^+^ T cells within tumors, a finding that correlates with favorable responses to ICBs therapies [53]. Taken together, increased LILRB1 expression may recruit SPP1‐ACP5^+^ macrophages to shape the TME. This process is likely reflected by enhanced CXCL13 secretion, which recruits additional CXCR5^+^ immune cells and ultimately leads to a reduced cancer cell fraction.

## 5 Limitations of the Study

First, additional clinical data should be collected and analyzed to delineate the signaling networks underlying LILRB1‐mediated changes in the TME. Second, the distinct roles of LILRB1 in immune cells versus tumor cells require further investigation. Third, efficacy evaluation models for PD‐1/PD‐L1‐targeted immunotherapy need to be constructed for HNSCC patients stratified by LILRB1 expression levels. Fourth, LILRB1 expression exhibits a significant positive correlation with immunosuppressive cells such as MDSCs and Tregs, except for immune‐activated cells. This observation suggests that high LILRB1 expression in HNSCC may also be associated with certain potential risks. Therefore, the dual functions of LILRB1 and its regulatory balancing effects require further exploration.

## 6 Conclusion

Collectively, LILRB1 is highly expressed in HNSCC samples and serves as a determinant of HNSCC prognosis. Specifically, LILRB1 influences prognosis, possibly by shaping the abundance and function of tumor‐reactive CD8^+^ T cells within the TME, a mechanism that may enhance the efficacy of immunotherapy. However, specific functional data and mechanistic insights regarding SPP1‐ACP5^+^ macrophages in HNSCC remain limited. Thus, additional experimental evidence is needed to validate the therapeutic potential of LILRB1‐targeted strategies in HNSCC.

## Author Contributions

**Shuai Chen:** conceptualization, data curation, funding acquisition, investigation, methodology, project administration, software, validation, visualization, writing – original draft, writing – review and editing. **Qiuwan Wu:** data curation, formal analysis, methodology, writing – original draft. **Shuo Gu:** data curation, formal analysis, methodology. **Yi Zhou:** formal analysis, funding acquisition, methodology, supervision. **Mingquan Cai:** data curation, funding acquisition. **Junhua Wu:** funding acquisition, investigation, validation, writing – original draft. **Jing He:** formal analysis. **Jingjing He:** data curation. **Juli Lin:** funding acquisition. **Zhicong Hong:** funding acquisition. **Binghuang Zhang:** conceptualization, funding acquisition, project administration, resources, writing – original draft, writing – review and editing. **Xianyang Luo:** conceptualization, funding acquisition, project administration, resources, supervision, writing – review and editing, writing – original draft. All authors have polished and approved the final manuscript.

## Funding

This work was supported by grants from the Natural Science Foundation of Fujian Province (2022J011363, 2024J011350, 2021J011358, 2024J011362, 2025J011429 and 2020J05302), Natural Science Basic Research Program of Xiamen City (3502Z20227339), Xiamen Medical and Health Guiding Project (3502Z20244ZD1032, 3502Z20244ZD1030, 3502Z20244ZD1041, 3502Z20244ZD2009 and 3502Z20244ZD1012) and the Science Foundation of the Fujian Provincial Commission of Health and Family Planning (2021GGB026).

## Ethics Statement

The study adhered to the principles of the Declaration of Helsinki. All the experiments were approved by the Medical Ethics Committee of the First Affiliated Hospital of Xiamen University (XMYY‐2021KYSB264). The Informed Consent Form was assigned to all cohorts in this study.

## Consent

The authors have nothing to report.

## Conflicts of Interest

The authors declare no conflicts of interest.

## Supporting information


**Data S1:** Supporting Information.

## Data Availability

Data were generated and will be made available upon request (Shuai Chen, email: chenshuai@xmu.edu.cn).
