# Supplementary material for: LILRB1 Is a Prognostic‐Related Biomarker Correlated With Immune Infiltration in Head–Neck Squamous Cell Carcinoma
Source: Cancer Med. 2026 Mar 25;15(3):e71727. doi: 10.1002/cam4.71727 (PMC13140679; doi:10.1002/cam4.71727)
Supplement: Supplementary file 1 — Data S1: Supporting Information. [file CAM4-15-e71727-s001.docx]

**Supplementary Figures and legends**

**
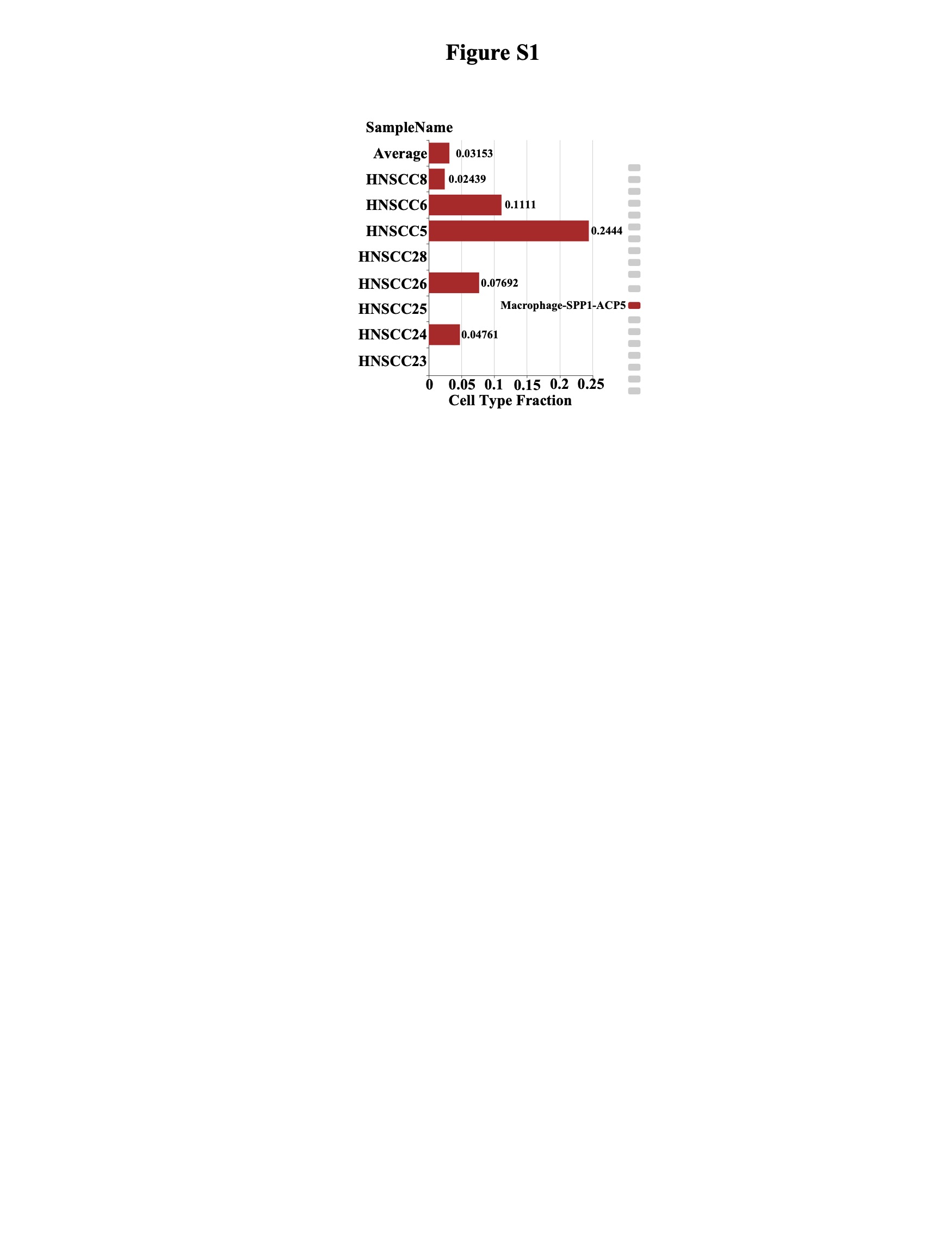
**

**Figure S1 The proportion of SPP1-ACP5^+^ macrophage in GSE103322 dataset.** There were 8 cases of HNSCC samples such as HNSCC8, HNSCC6, HNSCC5, HNSCC28, HNSCC26, HNSCC25, HNSCC24 and HNSCC23. In the single-cell sequencing results, SPP1-ACP5^+^ macrophage was found in 5 cases of all clinical samples, and the average proportion of SPP1-ACP5^+^ macrophages was 3.2% in all samples.


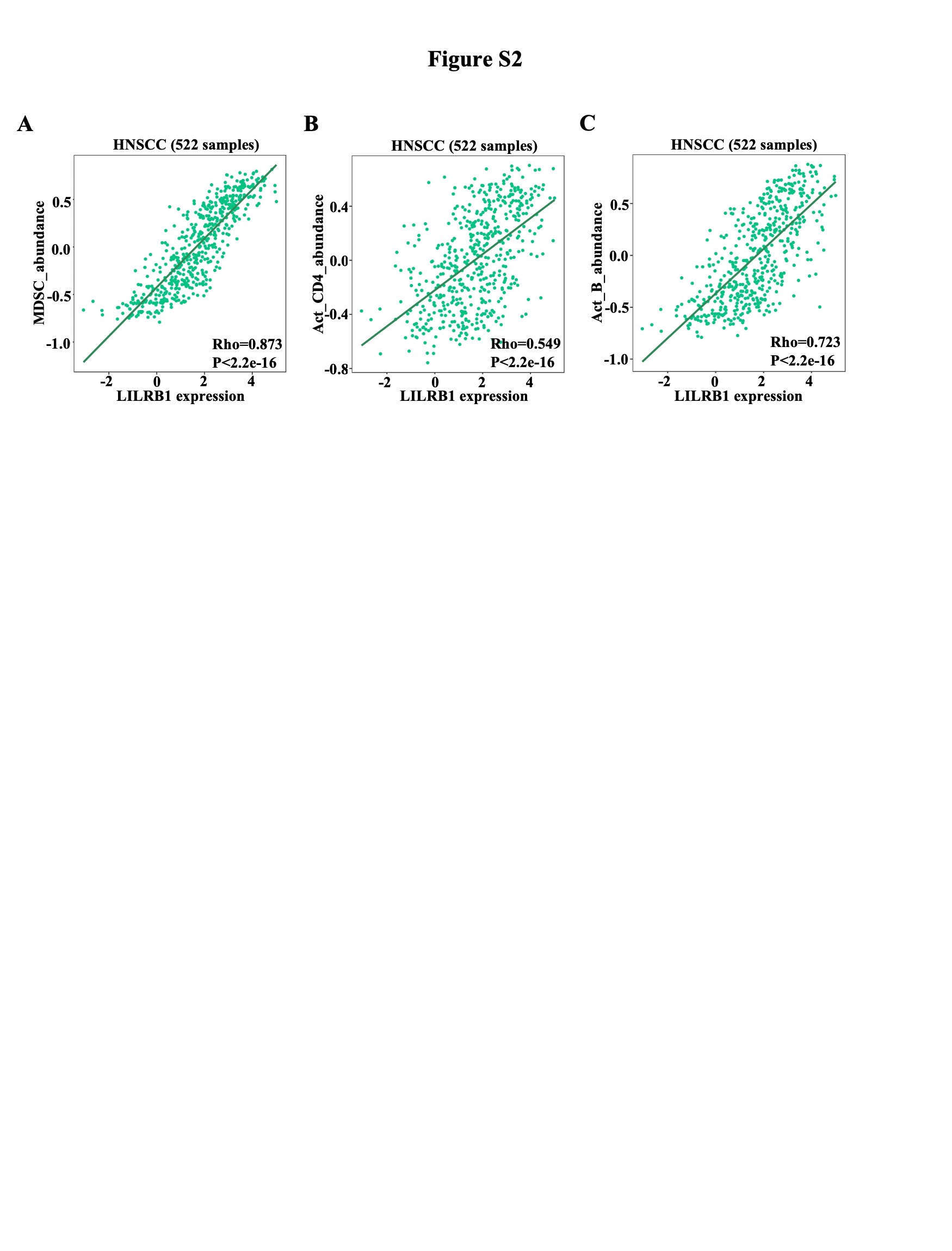


**Figure S2** **The positive-association between LILRB1 expression and immune cell abundance in HNSCC.** **(A-C)** Association between LILRB1 expression with myeloid-derived suppressor cell (MDSC) abundance (A), Act-CD4^+^ T cell abundance (B) and Act_B cell abundance (C) in HNSCC as analyzed by TIMER database. *P* < 0.001.


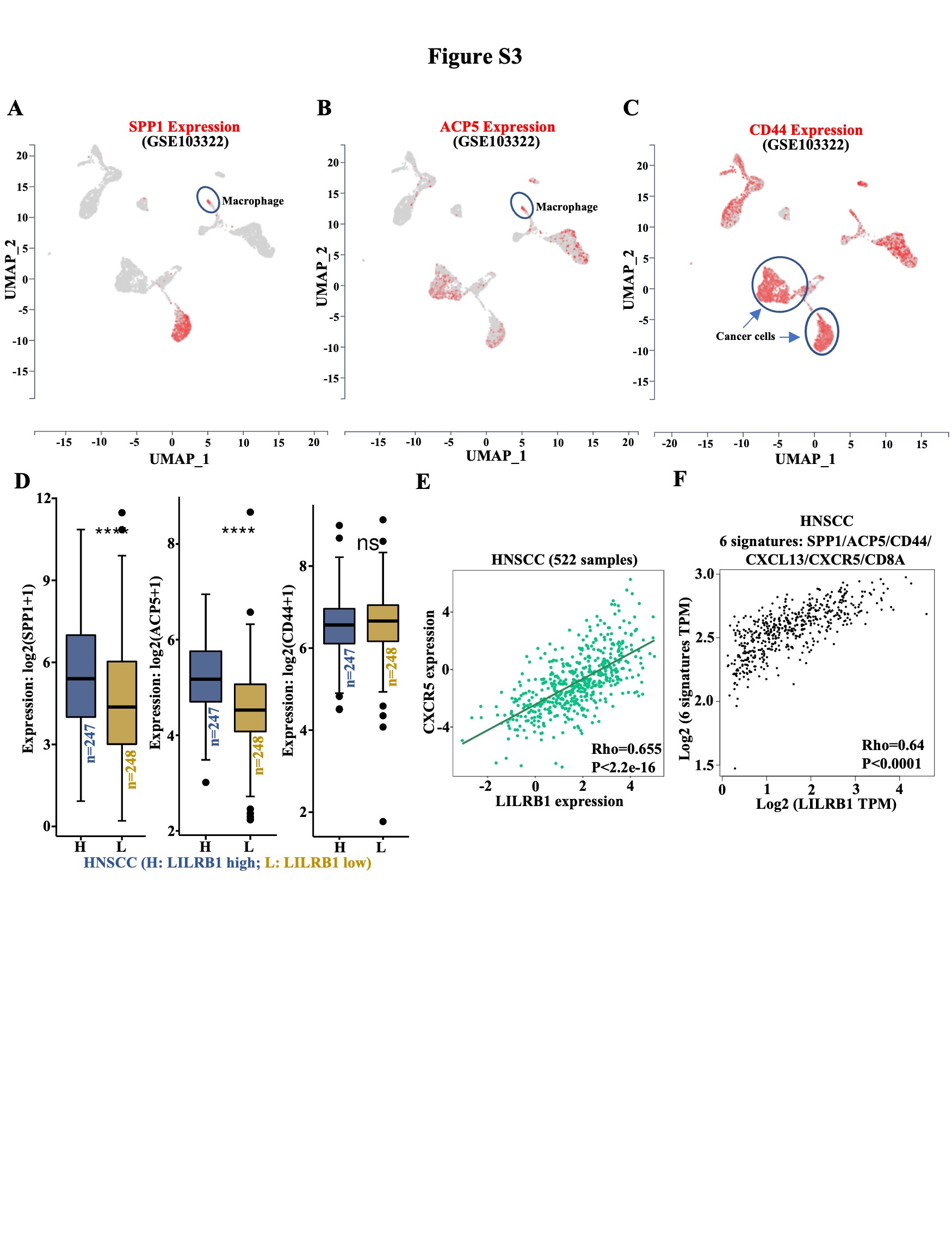


**Figure S3 The association among SPP1-CD44 axis, ACP5, CXCL13/CXCR5 axis, and CD8^+^ T cells in HNSCC using scRNA-seq datasets from open databases. (A-C)** In GSE103322 dataset, UMAP plot showing the expression distribution of SPP1 (left) (A), ACP5 (medium) (B), and CD44 (right) (C) in macrophage and cancer cell of 8 cases of HNSCC samples. **(D)** HNSCC patients were divided into two group including LILRB1^high^ (n = 247) and LILRB1^low^ (n = 248) according to LILRB1 expression status. Histogram visually displaying SPP1, ACP5, and CD44 expression difference in LILRB1^high^ and LILRB1^low^ HNSCC patients. **(E)** Association between LILRB1 expression and CXCR5 expression in HNSCC samples (n = 522) as analyzed by TIMER database. **(F)** Association between LILRB1 expression with six gene signatures (SPP1/ACP5/CD44/CXCL13/CXCR5/CD8A) in HNSCC as analyzed by GEPIA2. ****, *P* < 0.0001.


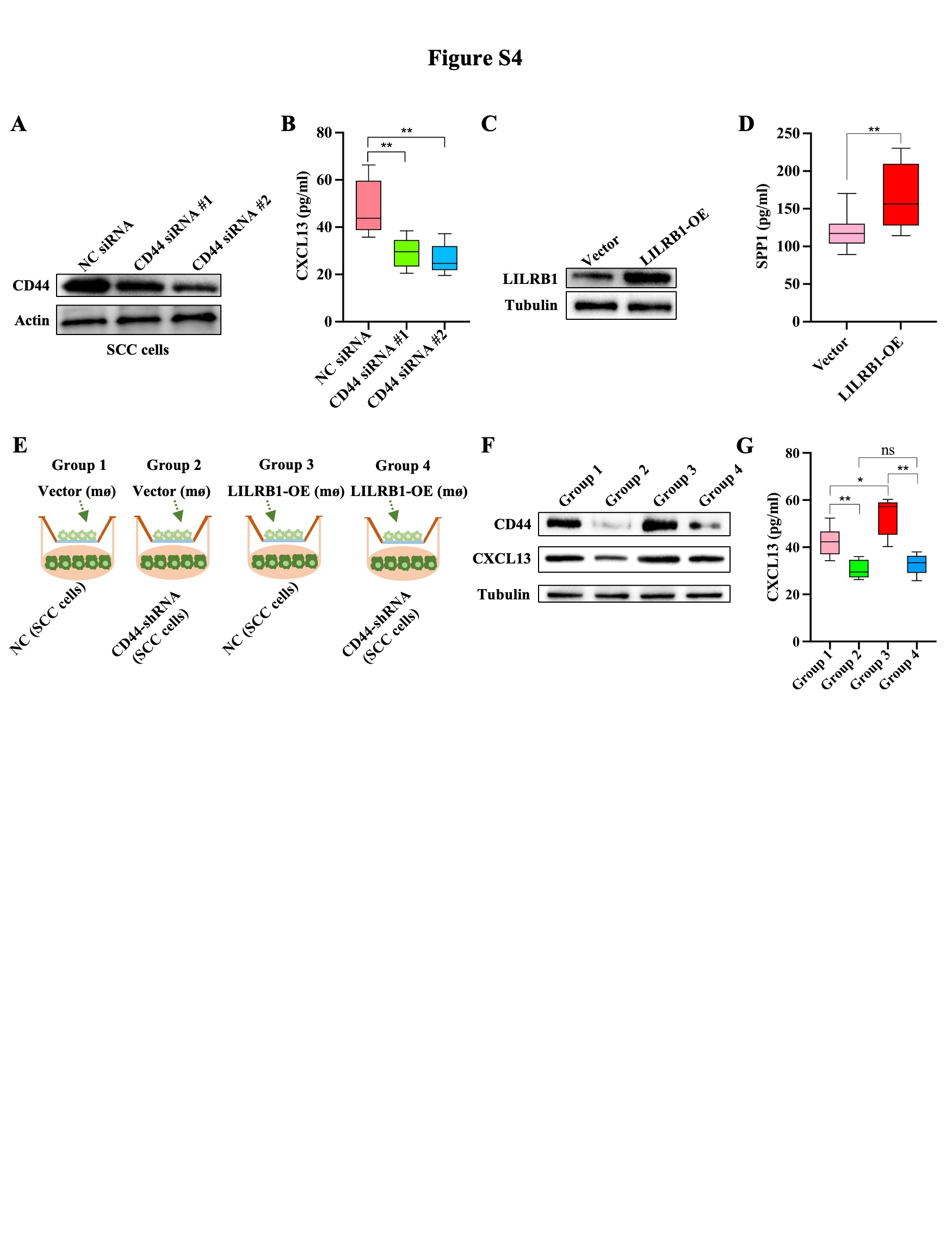


**Figure S4 Effects of CD44 on the interaction between macrophages and cancer cells. (A)** CD44 was knocked down in SCC7 cells and knockdown efficiency was verified using Western blotting. **(B)** THP-1 cells were cultured in the upper chamber and SCC cells were cultured in the lower chamber of transwell chamber. After 24 h of co-culture, CXCL13 production from SCC7 cells as determined by ELISA. **(C)** WB image presented protein expression of LILRB1 and Tubulin in control and LILRB1-overexpressing macrophages. **(D)** In LILRB1-overexpressing macrophages (THP-1 cells), SPP1 secretion was determined using ELISA. **(E-G)** Control and LILRB1 overexpressing THP-1 cells were seeded into the upper chamber of transwell and induced to completely differentiate into macrophages for 48 hours; SCC cells were seeded in the lower chamber at the same time (E). After continuous culture for 24 hours, the protein of tumor cells in the lower chamber and the supernatant were extracted to detect the expression of CD44/CXCL13 (F) and CXCL13 secretion of SCC cells (G). *, *P* < 0.05; **, *P* < 0.001.


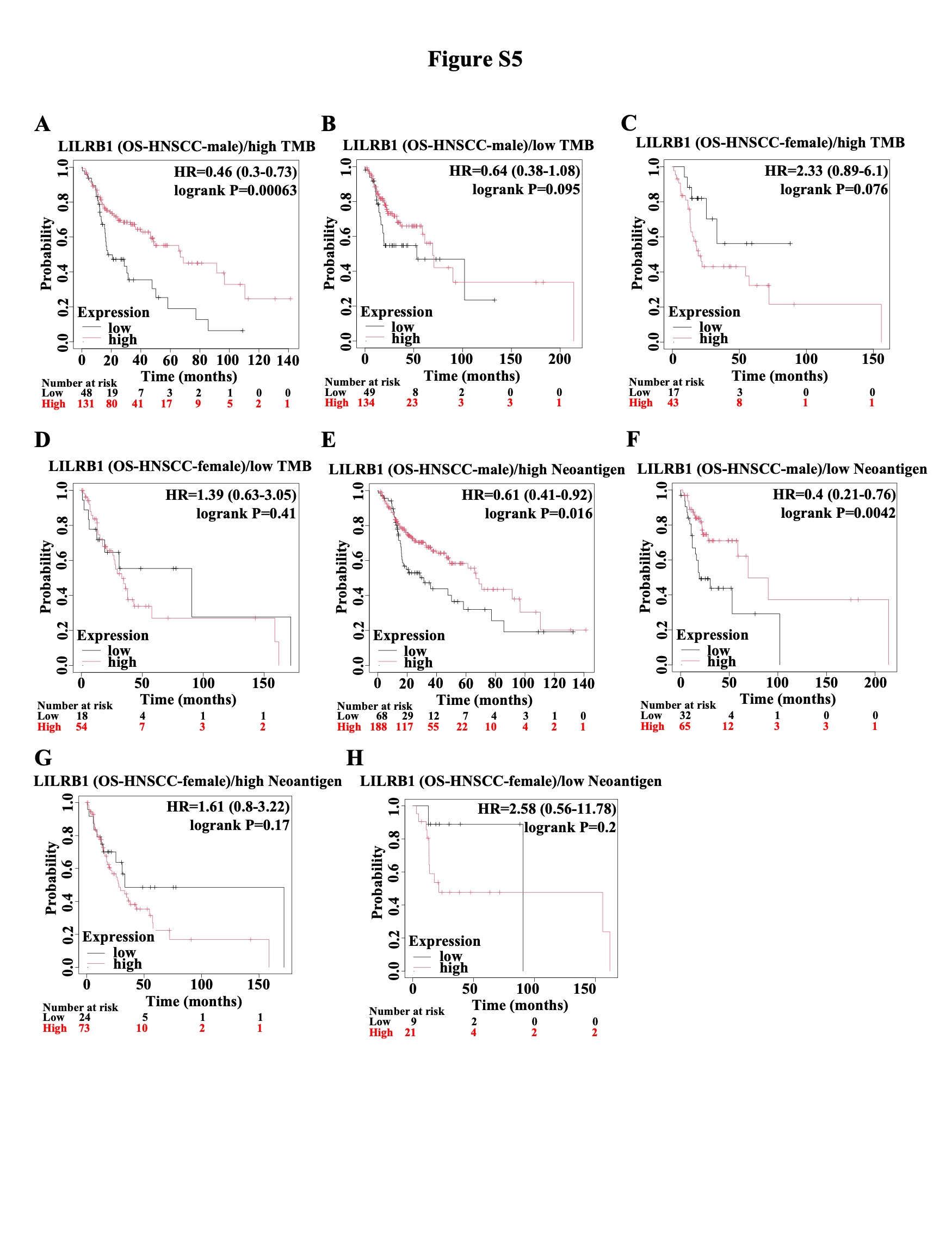


**Figure S5 Clinical prognosis analysis of LILRB1 expression in male/female HNSCC. (A-B)** Overall survival curve of differential LILRB1 expression in male HNSCC with high tumor mutation burden (TMB) (A) and low TMB (B). **(C-D)** Overall survival curve of differential LILRB1 expression in female HNSCC with high TMB (C) and low TMB (D). **(E-F)** Overall survival curve of differential LILRB1 expression in male HNSCC with high neoantigen load (E) and low neoantigen load (F). **(G-H)** Overall survival curve of differential LILRB1 expression in female HNSCC with high neoantigen load (E) and low neoantigen load (F).
